# Measuring the psychological drivers of participation in collective action to address violence against women in Mumbai, India

**DOI:** 10.12688/wellcomeopenres.15707.2

**Published:** 2020-06-29

**Authors:** Lu Gram, Suman Kanougiya, Nayreen Daruwalla, David Osrin

**Affiliations:** 1 University College London, Institute for Global Health, London, WC1N 1EH, UK; 2 SNEHA, Prevention of Violence Against Women, Mumbai, India

**Keywords:** violence against women, community, collective action, scale validation, India, urban health, gender

## Abstract

**Background: **A growing number of global health interventions involve community members in activism to prevent violence against women (VAW), but the psychological drivers of participation are presently ill-understood. We developed a new scale for measuring three proposed drivers of participation in collective action to address VAW in the context of urban informal settlements in Mumbai, India: perceived legitimacy, perceived efficacy, and collective action norms.

**Methods: **We did a household survey of 1307 men, 1331 women, and 4 trans persons. We checked for 1) social desirability bias by comparing responses to self-administered and face-to-face interviews, 2) acquiescence bias by comparing responses to positive and negatively worded items on the same construct, 3) factor structure using confirmatory factor analysis, and 4) convergent validity by examining associations between construct scores and participation in groups to address VAW and intent to intervene in case of VAW.

**Results:** Of the ten items, seven showed less than five percentage point difference in agreement rates between self-administered and face-to-face conditions. Correlations between opposite worded items on the same construct were negative (p<0.05), while correlations between similarly worded items were positive (p<0.001). A hierarchical factor structure showed adequate fit (Tucker-Lewis index, 0.919; root mean square error of approximation, 0.036; weighted root mean square residual, 1.949). Comparison of multi-group models across gender, education, caste, and marital status showed little evidence against measurement invariance. Perceived legitimacy, efficacy and collective action norms all predicted participation in groups to address VAW and intent to intervene in case of VAW, even after adjusting for social capital (p<0.05).

**Conclusion: **This is the first study to operationalize a measure of the psychological drivers of participation in collective action to address VAW in a low- and middle-income context. Our novel scale may provide insight into modifiable beliefs and attitudes community mobilisation interventions can address to inspire activism in similar low-resource contexts.

## Introduction

Worldwide, violence against women (VAW) is a critical public health problem with severe human, emotional, and economic costs ^1^. One form of VAW, intimate partner violence, affects 30% of women at least once in their lifetime, and is an important cause of mental, physical, sexual, and reproductive harm ^2^. International declarations including the United Nations Sustainable Development Goals and the Convention on the Elimination of all forms of Discrimination Against Women have committed national governments to eliminating VAW ^3^. However, our understanding of appropriate policies for achieving this is evolving.

Community mobilisation interventions have long been of interest to policymakers and practitioners as a means of addressing challenging societal and environmental barriers to achieving health ^4^. They can be defined as interventions in which local individuals collaborate with external agents in identifying, prioritising, and tackling the causes of ill-health based on principles of bottom-up leadership and empowerment ^5^. For example, interventions in South Africa and Uganda have trained volunteer activists to take action against violence, engaged community groups in reflection and action over unequal gender norms, and organised large-scale campaigns and marches ^6– 8^.

A key problem for the delivery of community mobilisation interventions is the extent to which they are able to successfully engage community members in activism ^9^. Given the risks associated with standing up to perpetrators of violence, community mobilisation interventions primarily seek to engage individuals in addressing VAW as part of coordinated efforts rather than as isolated actors ^6– 8^. Collective action – defined here as voluntary joint action by a group of people in pursuit of a shared goal ^10^ – becomes a particularly apt construct for exploring activism. However, participation in collective action poses unique theoretical problems for research and practice, because socially related individuals making decisions together behave differently from single individuals making isolated decisions about whether to take action against violence ^9^. Thus, collective action to address VAW overlaps with, but differs from the related concept of ‘bystander intervention’ ^11^ by emphasising intentional participation in a collective effort rather than ad hoc crisis response by individuals.

Promoting women’s capacity for collective action to address VAW also contributes to global policy commitments to advance gender equality and women’s empowerment ^12^. Researchers have extensively studied individualistic conceptions of agency and empowerment using measures of household decision-making power, spousal bargaining power, and access to material resources ^13– 16^. However, women’s collective agency and empowerment remain poorly understood, and few quantitative measures are available ^17^. Group participation ^18^ and social network structure ^19^ have been used in the past, but such proxies preclude researchers from separating out capacity for collective action from participation in such action. Groups and associations are often dynamic social structures, which form, disband or evolve based on perceived need ^20^, thus capacity for collective action may be just as important to track as actual participation.

Social scientists have long studied capacity for collective action for environmental and political causes ^21^ and proposed a range of psychological drivers of participation such action, which have yet to be widely applied to community mobilisation research in low- and middle-income countries. From a social psychology perspective, the main drivers are the perceived legitimacy of collective action, its perceived efficacy, and its relevance for community members’ social identity ^22^. From a sociologic and economic perspective, an important driver is the extent to which social norms reward or punish participation in collective action ^23^. We wanted to draw on these theories to develop a new scale for measuring drivers of participation in collective action to address VAW in a low-resource context and so obtain information on community members’ capacity for such action.

Our study was embedded in an ongoing cluster-randomized controlled trial of a complex community intervention to prevent violence against women in urban informal settlements (slums) in Mumbai, India ^24^. The primary outcomes were the prevalence of physical or sexual domestic violence and the prevalence of emotional or economic domestic violence, control, or neglect, both in the preceding 12 months. Secondary outcomes included non-partner sexual violence. The community mobilization intervention engaged community organisers in convening groups of women, men, and adolescents over a three-year period to address VAW on a platform of counselling, therapy, and legal services. Our research question addressed the extent to which it was possible to measure the psychological drivers of collective action against VAW in the context of urban informal settlements in Mumbai.

### Theoretical framework

**Figure 1.**
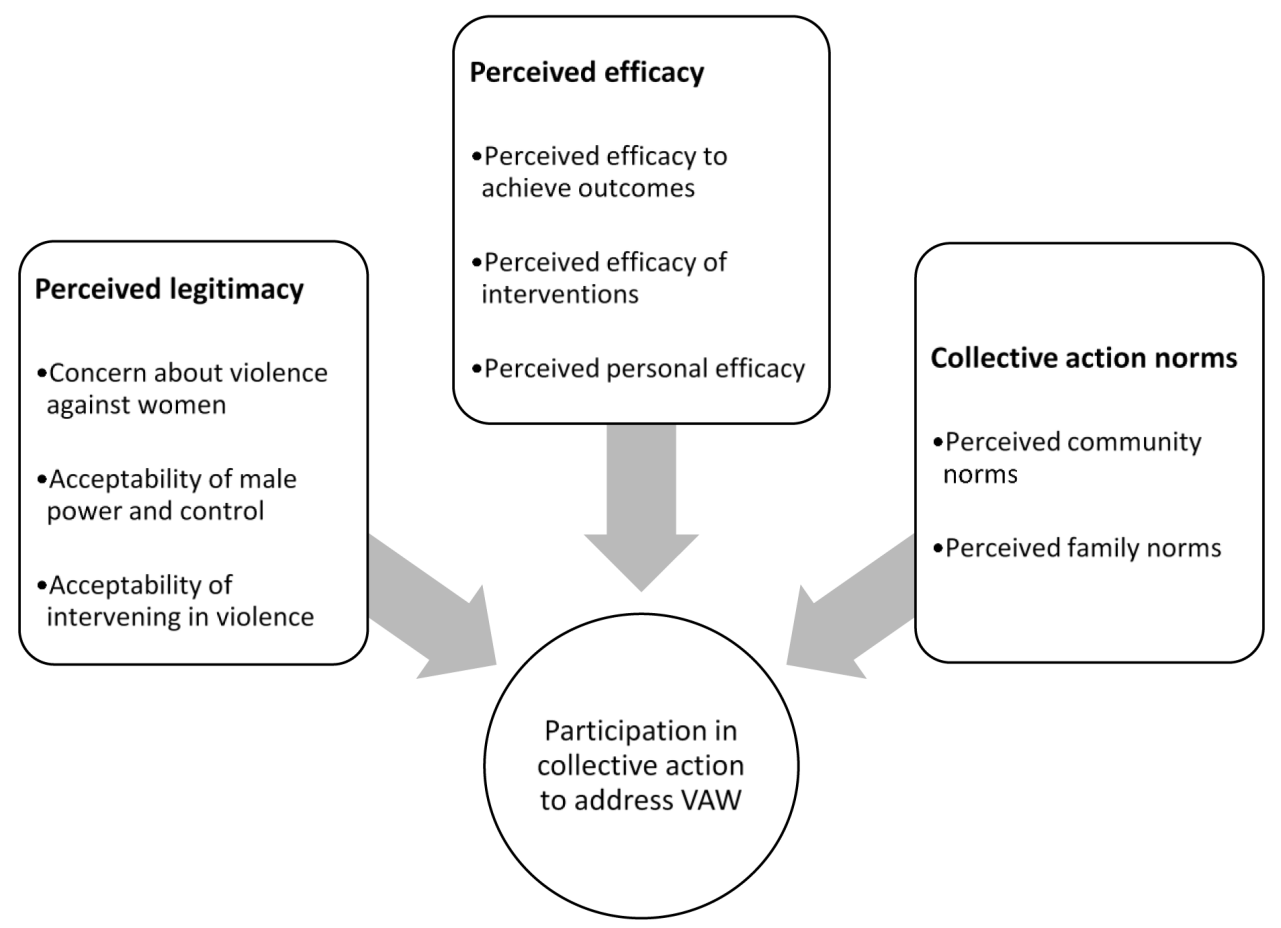
Theoretical framework. VAW, violence against women.

Figure 1 shows the overall theoretical framework for our measuring tool. We have discussed the general conceptual basis for applying collective action theory to community mobilisation elsewhere ^9^. Specifically for our context, community activism to prevent VAW may involve social dilemmas in which community members have an individual interest in abstaining from costly activism to change entrenched patriarchal norms perpetuating violence and letting others contribute, but no benefit is produced if nobody participates. To overcome such dilemmas, community members may be motivated through beliefs in the intrinsic rightness of participation in activism ^22^, beliefs that their own participation makes a difference ^22^, or beliefs that external rewards (or punishments) will ensue from participation (or non-participation) ^23^. To measure these beliefs, we examined the following constructs:

*Perceived legitimacy.* This construct refers to the extent to which action against VAW is seen as justified. It aligns with a number of theories positing that perceived grievance, injustice, or deprivation ^22^ motivate collective action for social change, while perceived justification for the status quo demotivates collective action ^25^. We divided the construct into three sub-constructs referring to respondent concern about VAW in general, acceptance of male power and control in the household, and beliefs about the acceptability of intervening in cases of VAW. These three sub-constructs are thought of as contributing to respondent feelings regarding the intrinsic rightness (or wrongness) of action against VAW.

*Perceived efficacy.* This refers to the extent to which participation in collective action is seen as an effective approach to addressing VAW. This construct aligns with theories positing that individuals need to feel their participation is potentially impactful before they judge it worthwhile ^22^. We divided it into three sub-constructs denoting respondents’ perceived efficacy to achieve specific outcomes (e.g. stop violence or get the police to take action), perceived efficacy of specific interventions (e.g. group discussions or marches and rallies), and the perceived contribution of their own participation.

*Collective action norms* refer to the extent to which community members expect others to approve or disapprove of them taking action to address VAW. This construct aligns with a tradition proposing that social norms imposing rewards or penalties for participation in collective action affect the ability of collectives to maintain high levels of participation ^23^. We divided it into two sub-constructs referencing respondents’ perceptions about the reaction of family and community members to their participation in action.

## Methods

### Setting

The NGO Society for Nutrition, Education and Health Action (SNEHA) runs a program on primary, secondary, and tertiary prevention of violence against women and children in Mumbai, India ^26^. The main beneficiaries of the program are residents of informal settlements, constituting 41% of Mumbai households ^27^. These are characterized by overcrowding, insubstantial housing, insufficient water and sanitation, lack of tenure, and hazardous location ^28^. Primary prevention is addressed through a combination of community group activities and resulting individual voluntarism. Secondary prevention includes local crisis response and psychological first aid by community organisers and referral to centres which provide counselling, legal, and psychotherapeutic support, with links to the police and medical, shelter, and social service providers. Tertiary prevention is provided primarily through referral to psychiatric and legal services.

### Indicator selection

We conducted a literature review of social movements, collective action and community mobilisation to inform choice of indicators ^4, 9^. We did not conduct a formal expert review. Items were selected and adapted from existing surveys where possible. We selected interview questions to ensure that different aspects of each theoretical construct were captured and each indicator had local relevance ( *Extended data*, Supplementary Table 1 ^29^ lists survey items for main and complementary measures in full). We selected indicators of perceived legitimacy from the Australian National Community Attitudes towards Violence Against Women Survey (ANCAVAWS) of 2009 ^30^. We measured perceived efficacy by adapting existing indicators of collective efficacy in community mobilisation research ^31^ and adding indicators from ANCAVAWS ^30^. We created our own items to measure collective action norms as no relevant existing measures were found, asking respondents what their family and community would think of them joining in activities to prevent VAW.

### Complementary measures

*Community social capital.* We selected indicators of social capital from the World Bank Social Capital Assessment Tool ^32^. Items represented a broad set of aspects of social capital including social networks, social cohesion, trust, cooperation, and altruism ^33^. The items asked if respondents knew their neighbours, trusted them, cooperated with them and could rely on them in emergencies. Following previous analyses of cluster-level social constructs ^34, 35^, we used multilevel factor analysis to generate estimates of factor scores ^36^. We modelled item values as arising from an individual’s perception of social capital using a 1-factor model (ordinal *α* = 0.855). These individual perceptions were aggregated into a measure of community social capital using a 1-factor model at the cluster level.

*Participation in groups to address VAW.* We adapted prior indicators of participation in groups to address specific issues in community mobilisation research ^31^. We first asked respondents whether they had participated in large-scale marches, rallies and protests, meetings organised by local community-based groups, or meetings of a non-governmental organisation in the past year. We then asked whether any of the community meetings or mass gatherings they had attended addressed VAW. If this was the case, we considered the respondent to have participated in a group to address VAW.

*Intent to intervene in cases of VAW.* We selected two indicators from the ANCAVAWS ^30^. The indicators asked how respondents would react if they were present when a woman was being physically assaulted by her partner. The first indicator asked how they would react if the woman was a stranger, the second if she was a family member or close friend. If the respondent indicated they would physically intervene or “say or do something else to try to help”, we classified them as intending to intervene in case of VAW.

### Piloting

We conducted iterative rounds of testing and modification of survey questions. LG developed survey items and ND and DO reviewed them. Qualitative researchers with extensive ethnographic experience in the same context also reviewed questions and translated them into Hindi and Marathi. LG conducted three unstructured group discussions with 14 interviewers about their understanding of the questions. LG observed 20 pilot interviews with local women and men, asking respondents clarifying questions where needed. These were not formal cognitive interviews ^37^, but organic interactions emerging from respondents providing short anecdotes or observations in response to survey items. If respondents only answered “yes” or “no” to our questions, LG probed for the reason behind their answer to stimulate discussion. Interviews were conducted face-to-face using smartphones running the CommCare application. At the end of this process, questions appeared to be well understood by respondents and could be asked within 45 minutes.

We were concerned about potential social desirability bias ^38^. Our survey involved respondents self-reporting motivation to take action against VAW to interviewers whom they knew came from an organization dedicated to eliminating VAW. Respondents might have felt social pressure to provide pleasing answers. To check for this, we designed a system that allowed respondents to self-administer survey questions: the interviewer would hand their smartphone to the respondent and ask them to press simple graphic icons to choose their answer without showing it to the interviewer, who would in turn read the questions aloud. The smartphone application chose 1 in 7 respondents randomly to receive this type of interview. Given its logistically onerous nature, we only tested it on questions about perceived efficacy to achieve specific outcomes, perceived efficacy of specific interventions and collective action norms.

We were also concerned about acquiescence bias ^38^. Respondents might feel pressured to agree to items regardless of content to avoid saying ‘no’ to the interviewer. Respondents might also agree with items without trying to properly understand them to finish the interview faster. To check for this, we ensured the survey contained both positively and negatively worded questions. If respondents agreed with questions without considering their meaning, they would agree to everything, including survey items making opposite statements. We tested this on questions about collective action norms.

### Data collection

Between December 2017 and December 2019, we carried out a baseline survey of community attitudes to VAW in households across 54 informal settlement clusters. Each cluster contained about 500 households. Clusters were in four large urban informal settlements, chosen for their vulnerability, low risk of rehabilitation, low coverage by organisations working to address VAW, and low proportion of rental tenancies. From a random starting point in each cluster, 16 investigators selected 25 women and 25 men aged 18 to 65 years – a single interviewee per household – by visiting households sequentially. We thus obtained approximately 50 interviews per cluster. Participants were enrolled in person. Inclusion criteria were that respondents should fall into these age groups and should provide signed consent.

The initial baseline survey comprised questions on attitudes to gender roles, gender equality, VAW, and bystander intervention, as described in our protocol ^24^. Questions on action to address VAW were added later, resulting in 92 respondents missing data on these questions. After dropping these (3%), the final sample size was 2642, of whom 1307 were cis men, 1331 cis women, and 4 trans women. Although there is currently no consensus method for determining sample sizes for scale validation ^39^, our sample size far exceeds the recommended minimum acceptable thresholds for factor analysis of 300 participants by Comrey and Lee ^40^ and 20 per survey item by Kline ^41^ (given we have 27 survey items).

We also randomised a calendared subgroup of 1899 respondents to receive either the self-administered or the face-to-face interview from June 2018 to December 2019. In total, 247 received the self-administered survey (13%) and 1652 received the face-to-face interview (87%). Interviews were conducted after provision of participant information sheets and signed consent. There was no requirement that the interview be private as the questions were not deemed sufficiently sensitive to put people at risk for answering them.

### Data analysis

***Item validity.*** We investigated item validity by checking for acquiescence and social desirability bias. To check for acquiescence bias, we compared responses to positively and negatively worded items on collective action norms using tetrachoric correlation ^42^. We chose tetrachoric correlation as items were binary. A well-performing scale would show negative correlation between positively and negatively worded items for a construct. To check for social desirability bias, we compared answers to self-administered and interviewer-administered questions using Pearson chi-squared tests. If bias was absent, we would see little difference. We further checked for differential impact of self-administered interviews on response patterns by gender. We ran separate logistic regression models for each item against administration mode interacted with gender using robust variance estimators to account for clustering.

***Construct validity.*** We investigated construct validity ^43^ using categorical confirmatory factor analysis, comparing four different factor structures in order of decreasing model restrictiveness:

1. A unidimensional model relating all items to a single factor.
2. A three-dimensional model relating items directly to the three main constructs: perceived legitimacy, perceived efficacy and collective action norms.
3. A hierarchical model relating items to eight first-order factors representing the eight sub-constructs from our theoretical framework (see Figure 1). These first-order factors loaded onto three second-order factors representing our three main constructs.
4. An eight-dimensional model relating items to eight first-order factors as in Model 4, but without the three second-order factors present.

We used the Tucker-Lewis index (TLI) and the root mean square error of approximation (RMSEA) to do this ^44^. For the TLI, a good fit was indicated by a value greater than 0.95, a poor fit by a value less than 0.90, and an adequate fit by a value in between. For the RMSEA, a good fit was indicated by a value less than 0.06, a poor fit by a value greater than 0.08, and an adequate fit by a value in between ^44^. We also computed weighted root mean square residual (WRMR) for which a good fit is usually indicated by a value less than 1.0 ^44^. However, the cut-off value of 1.0 is known to be overly sensitive to minor model deviations for sample sizes above 1,000, so WRMR is considered ‘experimental’ ^45^.

We assessed internal consistency using ordinal *α*, a modified version of Cronbach’s *α* for ordinal data ^46^. We did not assess test-retest reliability. Past experience in our context – namely vulnerable, low-literacy populations living in informal settlements in Mumbai – has shown that returning to re-interview respondents can create problems, as respondents believe their anonymity has been breached by us being able to track them down for a re-interview.

Finally, we assessed measurement invariance across gender, education, caste, and marital status by comparing fit statistics (TLI, RMSEA, and WRMR) for models of configural invariance, invariance of first-order factor loadings, invariance of second-order factor loadings, and invariance of both ^47^. For the purpose of this comparison, we used binary codes for education (any versus no education), caste (Open/General versus other caste groups), and marital status (currently married versus not currently married). We did not conduct *χ* ^2^ tests as these are known to be overly dependent on sample size, which creates oversensitivity to small deviations from H _0_ in large samples and spurious sensitivity to differences in sample size between comparison groups in measurement invariance tests ^48^.

***Criterion validity.*** We examined criterion validity ^43^ by checking for convergent validity. We calculated empirical Bayes estimates for the each construct in our preferred model from the prior factor analysis ^49^. We fitted separate generalized structural equation models for each factor with paths from social capital to the factor, from the factor to a behaviour-related outcome, and from social capital directly to the same outcome. We examined three outcomes: participation in groups to address VAW, intent to intervene in case of violence against a stranger, and intent to intervene in case of violence against a family member. We adjusted for clustering using robust standard errors. We modelled all outcomes as binary responses linked to predictors via a logit link. By checking whether each factor was associated with each outcome, even after adjusting for social capital, we obtained evidence for convergent validity. In case our preferred model was a hierarchical model, we fitted generalized structural equation models for 2 ^nd^-order factors, but only logistic regression models for 1 ^st^-order factors, adjusting for social capital; in such a case, associations with 2 ^nd^-order factors were our primary interest.

### Missing data

In total, 30% of respondents did not know the answer to at least one question on collective action. These respondents were slightly more likely to be younger, unmarried, Muslim, of non-scheduled caste, uneducated, and unemployed, although chance could only be ruled out for age, caste, and educational differences (p<0.05; see *Extended data*, Supplementary Table 2 ^29^). In 86% of these cases, respondents were able to respond to at least 24 out of 27 questions and the proportion of “don’t know” answers never exceeded 8% for any individual item.

We therefore used complete-case analysis for item validity. To correct for bias in assessing criterion validity, we imputed factor scores in Empirical Bayes estimates in which items on collective action to address VAW were missing. We used weighted least squares estimation under a missing at random conditional on observables assumption ^50^, modelling factor scores as dependent on age, marital status, religion, caste, education and employment.

### Software

Factor analysis was carried out in MPlus 7.11; all other analyses used Stata/SE 15.1. For replication purposes, R is an open access alternative.

### Ethics

The trial in which the data were collected is registered with the Controlled Trials Registry of India ( CTRI/2018/02/012047) and ISRCTN ( ISRCTN84502355). Ethical approval was granted by the UCL Research Ethics Committee (3546/003, 27/09/2017) and by PUKAR (Partners for Urban Knowledge, Action, and Research) Institutional Ethics Committee (25/12/2017). We had gatekeeper consent for inclusion of clusters in the trial. Interviewers provided a participant information sheets to respondents, discussed the nature of the interview, and obtained signed consent.

## Results

### Descriptive data

Table 1 shows the demographic profile of the sample. Most respondents were 25–44 years’ old and married. Male respondents were more likely to be unmarried than female respondents. The majority of residents identified as Hindu or Muslim and belonged to a general or scheduled caste. In total, 43% of women and 32% of men did not have a high-school education, while 78% of women and only 24% of men had no employment. Employed women were substantially more likely than men to do home-based piecework. De-identified, individual-level results are available as *Underlying data* ^29^.

**Table 1.** Demographic profile of respondents.

	*Women* *and trans* *people*		*Men*		*Test of* *difference*
**Age**	n	%	n	%	p-value
<24 years	282	21%	317	24%	<0.001
25–34 years	535	40%	377	29%	
35–44 years	331	25%	283	22%	
45+ years	187	14%	330	25%	
**Marital status**					
Unmarried	152	11%	401	31%	<0.001
Married	1092	82%	888	68%	
Separated/ divorced/ widowed	88	7%	17	1%	
Other	3	0%	1	0%	
**Primary** **language**					
Marathi	463	35%	439	34%	0.353
Hindi/Urdu	775	58%	788	60%	
Other	97	7%	80	6%	
**Religion**					
Hindu	786	59%	805	62%	0.048
Muslim	446	33%	372	28%	
Christian	14	1%	20	2%	
Sikh	3	0%	3	0%	
Buddhist/Neo- Buddhist	85	6%	79	6%	
Did not want to say	1	0%	28	2%	
**Caste**					
Open/General	788	59%	708	54%	0.003
OBC	253	19%	285	22%	
Scheduled caste (SC)	218	16%	232	18%	
Scheduled tribe (ST)	18	1%	25	2%	
None of these	57	4%	29	2%	
Did not want to say	1	0%	28	2%	
**No. household** **members**					
1	6	0%	10	1%	0.020
2	82	6%	118	9%	
3	221	17%	213	16%	
4+	1026	77%	966	74%	
**Duration** **of stay in** **Mumbai**					
0–4 years	51	4%	53	4%	0.010
5–14 years	177	13%	145	11%	
15–24 years	261	20%	205	16%	
25+ years	840	63%	896	69%	
Did want to say	0	0%	0	0%	
**Education**					
No formal education	135	10%	74	6%	<0.001
Primary (1–5th standard)	155	12%	144	11%	
Middle (6–8th standard)	286	21%	199	15%	
High school (9– 10th standard)	365	27%	395	30%	
Senior school (11–12th standard)	210	16%	226	17%	
Undergraduate or higher	184	14%	266	20%	
Other	0	0%	3	0%	
**Type of** **employment**					
No employment	1,035	78%	308	24%	<0.001
Home-based earnings	176	13%	104	8%	
House maid, sweeper, construction or agriculture	8	1%	57	4%	
Vendor job	8	1%	62	5%	
Shop, parlour, saloon owner	1	0%	116	9%	
Driver-Taxi/ auto/cab/bus	30	2%	31	2%	
Job/service	64	5%	550	42%	
Salaried job, consultant, executive	10	1%	57	4%	
Other	3	0%	22	2%	
**Monthly** **earned income**					
Unpaid	18	6%	42	4%	<0.001
<INR 1,000	57	19%	4	0%	
INR 1,001– 10,000	196	65%	350	35%	
INR 10,001– 100,000	29	10%	602	60%	
INR 100,001+	0	0%	0	0%	

We found high levels of social cohesion in our communities, as 81–93% of respondents reported feeling at home in their neighbourhood or getting along with neighbours (see *Extended data*, Supplementary Table 3 ^29^). 71–91% agreed that people came together to keep the neighbourhood free from crime or solve issues such as disruptions to the water supply. However, levels of trust were lower, as 58–61% stated that their neighbours could not be trusted or only looked out for themselves. 56% of women and 46% of men stated they did not recognise most people in their neighbourhood (p<0.001 for a gender difference).

### Item validity

***Social desirability bias.*** Table 2 shows item responses on the constructs of perceived efficacy to achieve specific outcomes, perceived efficacy of specific interventions, and collective action norms where questions were either self-administered or entered by the interviewer. Due to our large sample size, we found statistically significant differences for some items, even if these seem small. For example, the proportion of respondents disagreeing with the item “together you can persuade families to support women facing domestic violence” only rose from 2% to 5% in the self-administered condition (p<0.001). Of the ten items, seven showed less than five percentage points difference in the proportion of respondents agreeing with the item between self- and interviewer-administered conditions.

**Table 2.** Comparing self-administered with face-to-face survey responses.

	*Self-administered survey*			*Face-to-face survey*			*Test of* *difference*
	Generally agree	Generally disagree	Don't know	Generally agree	Generally disagree	Don't know	p-value
*Perceived effectiveness to achieve specific* *outcomes*							
In your neighbourhood, you can stop domestic violence by working together	87% (214)	10% (25)	3% (8)	86% (1423)	12% (192)	2% (37)	0.530
By working together, you can persuade the police to take action against domestic violence	87% (215)	10% (24)	3% (8)	87% (1429)	11% (187)	2% (36)	0.480
Together you can persuade families to support women facing domestic violence	93% (230)	5% (13)	2% (4)	97% (1607)	2% (41)	0% (4)	0.003
*Perceived effectiveness of* *specific interventions*							
Do you think the following activities are effective in stopping violence against women…							
- Group meetings and discussions	88% (218)	6% (14)	6% (15)	91% (1500)	7% (122)	2% (30)	0.001
- Marches, rallies or street theatre	81% (200)	14% (35)	5% (12)	80% (1318)	18% (301)	2% (33)	0.017
- Sit-ins, blockages or strikes	37% (92)	54% (134)	9% (21)	31% (509)	64% (1052)	6% (91)	0.012
	*Self-administered survey*			*Face-to-face survey*			*Test of* *difference*
	Generally agree	Generally disagree	Don't know	Generally agree	Generally disagree	Don't know	p-value
*Perceived community norms*							
People in your neighbourhood approve of you joining activities to stop violence against women	75% (185)	15% (36)	11% (26)	80% (1316)	15% (252)	5% (84)	0.005
People in your neighbourhood would mock you for joining activities to stop violence against women	40% (99)	49% (120)	11% (28)	40% (664)	51% (843)	9% (145)	0.808
You would be embarrassed to say in public that you work to prevent violence against women	11% (27)	87% (214)	2% (6)	4% (67)	95% (1576)	1% (9)	<0.001
*Perceived family norms*							
Your family members approve of you joining activities to stop violence against women	79% (196)	15% (38)	5% (13)	82% (1352)	16% (259)	2% (41)	0.044
Your family members consider activities to stop violence against women opposed to their own values	34% (83)	64% (159)	2% (5)	22% (356)	76% (1258)	2% (38)	0.002
Your family members consider spending one hour a week to stop violence against women a waste of your time	25% (62)	71% (176)	4% (9)	20% (327)	78% (1286)	2% (39)	0.053
Your family members consider activities to stop violence against women prestigious work	76% (188)	16% (39)	8% (20)	77% (1280)	18% (298)	4% (74)	0.134

However, the proportion agreeing that “your family members consider activities to stop VAW opposed to their own values” rose from 22% in the face-to-face to 34% in the self-administered condition (p=0.002). The proportion agreeing that “you would be embarrassed to say in public that you work to prevent VAW” rose from 4% to 11% (p<0.001), while the proportion disagreeing that sit-ins, strikes, and blockades are effective in preventing VAW fell from 64% to 54% (p<0.001). These items might have been particularly sensitive to social desirability bias.

The proportion of respondents providing “don’t know” answers generally increased by 1–4 percentage points the self-administered survey condition (p<0.05). However, for the item “People in your neighbourhood approve of you joining activities to stop violence against women”, the proportion of “don’t know” answers increased by fully 6 pp (p=0.005). With respect to gender differences in social desirability, we found no evidence for an interaction with gender in all indicators (p>0.05) except for the item “Your family members approve of you joining activities to stop violence against women” (p=0.008), where the self-administered interview had a clear effect on men’s odds of agreeing (OR 0.51, p=0.037, 95% CI 0.27-0.97), but not women’s odds (OR 1.36, p=0.20, 95% 0.85-2.19).

***Acquiescence bias.*** Table 3 shows pairwise tetrachoric correlations of items for collective action norms. For all items except one, we found high negative correlations between items of opposite polarity within the same sub-construct, ranging from -0.75 to -0.63. For example, the correlation between the item “people in your neighbourhood approve of you joining activities to stop VAW” and “people in your neighbourhood would mock you for joining activities to stop VAW” was -0.63. The correlation between the item “you would be embarrassed to say in public that you work to prevent VAW” and the item “people in your neighbourhood approve of you joining activities to stop VAW” was only -0.12. However, it was still negative with sufficient evidence to reject a null hypothesis of zero correlation (p=0.016).

**Table 3.** Pairwise correlations between items on collective action norms. N=2,642.

	People in your neighbourhood approve of you joining activities to stop VAW	People in your neighbourhood would mock you for joining activities to stop VAW	You would be embarrassed to say in public that you work to prevent VAW	Your family members approve of you joining activities to stop VAW	Your family members consider activities to stop VAW opposed to their own values	Your family members consider spending one hour a week to stop VAW a waste of your time	Your family members consider activities to stop VAW prestigious work
People in your neighbourhood approve of you joining activities to stop VAW	-						
People in your neighbourhood would mock you for joining activities to stop VAW	-0.63	-					
You would be embarrassed to say in public that you work to prevent VAW	-0.12	0.29	-				
Your family members approve of you joining activities to stop VAW	0.55	-0.34	-0.24	-			
Your family members consider activities to stop VAW opposed to their own values	-0.29	0.46	0.47	-0.73	-		
Your family members consider spending one hour a week to stop VAW a waste of your time	-0.33	0.43	0.47	-0.75	0.81	-	
Your family members consider activities to stop VAW prestigious work	0.46	-0.32	-0.20	0.83	-0.65	-0.70	-

VAW, Violence against women.

Except for one item, correlations between items of the same polarity within the same sub-construct were also high, ranging from 0.81 to 0.83. Correlations between items across sub-constructs were smaller in magnitude, ranging from -0.34 to 0.55. The correlation between the item “you would be embarrassed to say in public that you work to prevent VAW” and the item “people in your neighbourhood would mock you for joining activities to stop VAW” was only 0.29. However, it was still positive with strong evidence to reject a null hypothesis of zero correlation (p<0.001). These results suggest that, overall, respondents were not simply agreeing with all survey items regardless of their content.

### Construct validity

Table 4 shows the results of confirmatory factor analysis, which indicated a poor fit for the unidimensional and three-factor models (TLI<0.9, RMSEA>0.05, WRMR>1) and an adequate fit for the hierarchical and eight-factor models (TLI>0.9, RMSEA<0.05, WRMR>1). There was little statistical reason to favour one of the two latter models. The TLI and RMSEA for both were nearly identical, although the WRMR for the eight-factor morel was slightly better than that of the hierarchical model (1.949 vs. 1.837). We chose the hierarchical model to assess criterion validity, as it exhibited greater parsimony in the number of model parameters and was more consistent with our theoretical framework.

**Table 4.** Fit statistics for different factor structures modelling drivers of collective action.

	TLI	RMSEA	WRMR
**Model 1:** Unidimensional model	0.573	0.082	4.159
**Model 2:** 3-factor model	0.818	0.053	2.832
**Model 3:** Hierarchical model	0.919	0.036	1.949
**Model 4:** 8-factor model	0.915	0.037	1.837

TLI, Tucker-Lewis index; RMSEA, root mean square error of approximation; WRMR, weighted root mean square residual.

**Figure 2.**
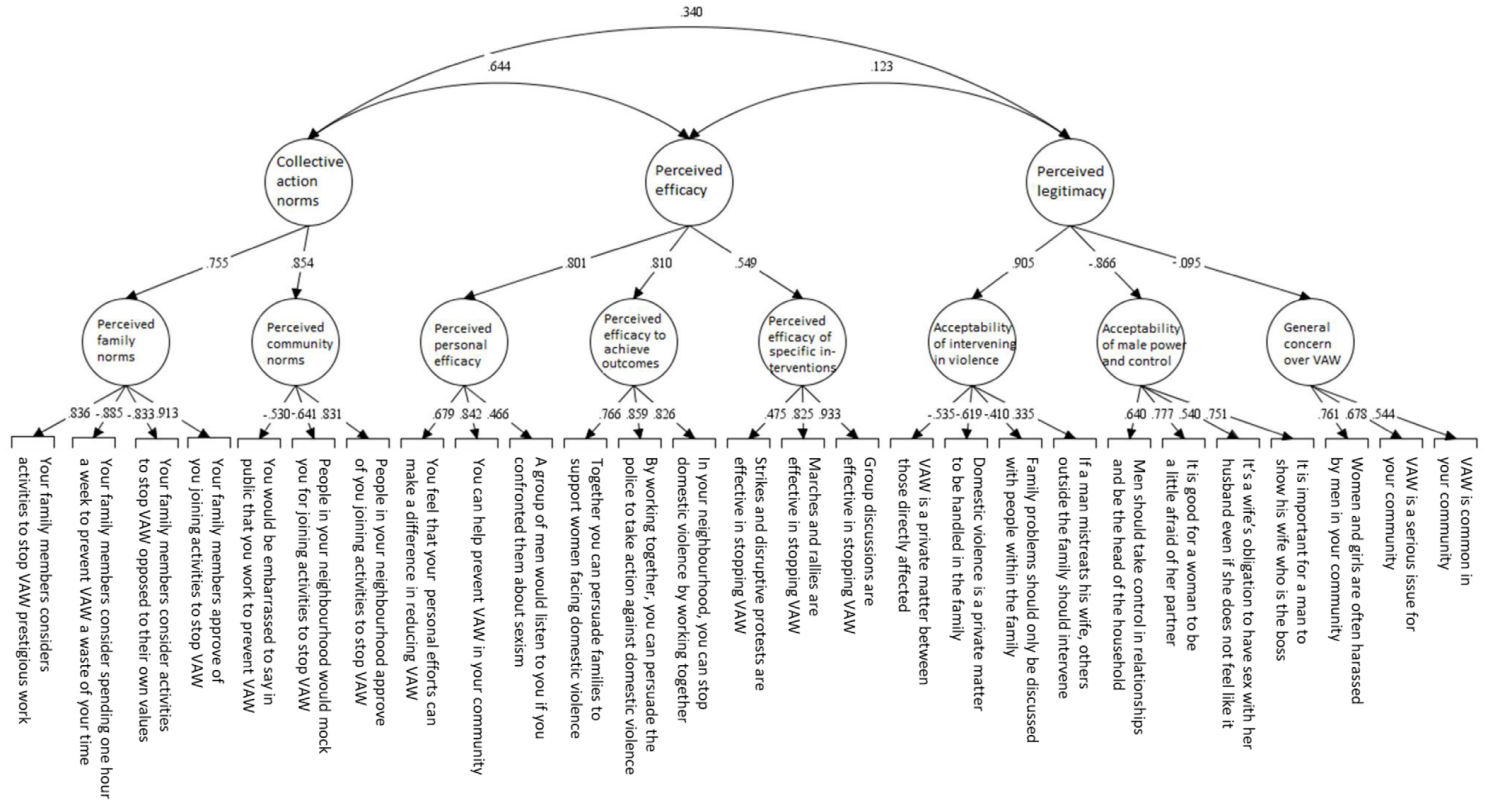
Factor loadings and correlations for higher-order model of drivers of collective action. All factor loadings and correlations are statistically significant at p<0.001. The three higher-order constructs collective action norms, perceived efficacy, and perceived legitimacy have been standardised to mean 0 and standard deviation 1. VAW, violence against women.

Figure 2 shows the factor loadings and correlations from the hierarchical model. All loadings and correlations were highly statistically significant (p<0.001). All except one were positive and negative in expected directions. For example, the loading on ‘if a man mistreats his wife, then others should intervene’ was positive (0.335), while all loadings on all other items for the same sub-construct were negative (≤-0.410). This made sense as the other items expressed the opposite attitude, that it was inappropriate to intervene in cases of violence. However, the sub-construct ‘concern for VAW’ loaded weakly on its parent construct ‘perceived legitimacy’ (-0.095) compared to sub-constructs ‘acceptability of male power and control’ (-0.866) and ‘acceptability of intervention in cases of VAW’ (0.905). This indicates, ‘concern for VAW’ is better considered as falling into a separate class of construct of its own, as opposed to sharing a family resemblance to the other two sub-constructs.

Table 5 shows ordinal alphas for main and sub-constructs. We found generally high levels of internal consistency for both main and sub-constructs considering the small number of items per construct. Ordinal alphas for collective action norms (0.874) and perceived efficacy (0.831) were high, even if sub-constructs community norms (0.574) and personal efficacy (0.658) had moderately low scores. The legitimacy domain had a moderately low score (0.694), as did sub-constructs acceptability of intervening in violence (0.499) and general concern over VAW (0.662).

**Table 5.** Ordinal alphas for main and sub-constructs.

Main construct	Sub-construct	Ordinal α
Perceived legitimacy (ordinal α = 0.694)	Concern over VAW	0.662
	Acceptability of male power and control	0.749
	Acceptability of intervening in violence	0.499
Perceived efficacy (ordinal α = 0.831)	Personal efficacy	0.658
	Perceived efficacy to achieve outcomes	0.801
	Perceived efficacy of specific interventions	0.765
Collective action norms (ordinal α = 0.874)	Perceived community norms	0.574
	Perceived family norms	0.915

We did not find strong evidence against measurement invariance ( Table 6). Comparing fit across models accounting for gender, education, caste, and marital status, both WRMR and TLI decreased slightly as constraints on factor loadings were lifted, while RMSEA stayed relatively constant. The decrease in WRMR indicates better fit for models allowing for heterogeneity across groups, but the decrease in TLI indicates a worse fit, possibly due to lower parsimony in the unconstrained models. Given these results, we did not see a need for separate measurement models by gender or demographic group.

**Table 6.** Comparing multi-group measurement models.

		*Gender*	*Education*	*Caste*	*Marital status*
*Configural invariance only*	*TLI*	*0.915*	*0.919*	*0.918*	*0.918*
	*RMSEA*	*0.035*	*0.031*	*0.035*	*0.034*
	*WRMR*	*2.160*	*2.158*	*2.151*	*2.136*
*Invariant first-order factor loadings*	*TLI*	*0.918*	*0.924*	*0.921*	*0.920*
	*RMSEA*	*0.034*	*0.030*	*0.034*	*0.033*
	*WRMR*	*2.172*	*2.173*	*2.162*	*2.169*
*Invariant second-order factor loadings*	*TLI*	*0.918*	*0.923*	*0.921*	*0.921*
	*RMSEA*	*0.034*	*0.030*	*0.034*	*0.033*
	*WRMR*	*2.182*	*2.166*	*2.169*	*2.156*
*Both first- and second-order factor loadings invariant*	*TLI*	*0.921*	*0.927*	*0.924*	*0.923*
	*RMSEA*	*0.034*	*0.030*	*0.034*	*0.033*
	*WRMR*	*2.200*	*2.182*	*2.182*	*2.189*

### Criterion validity

**Figure 3.**
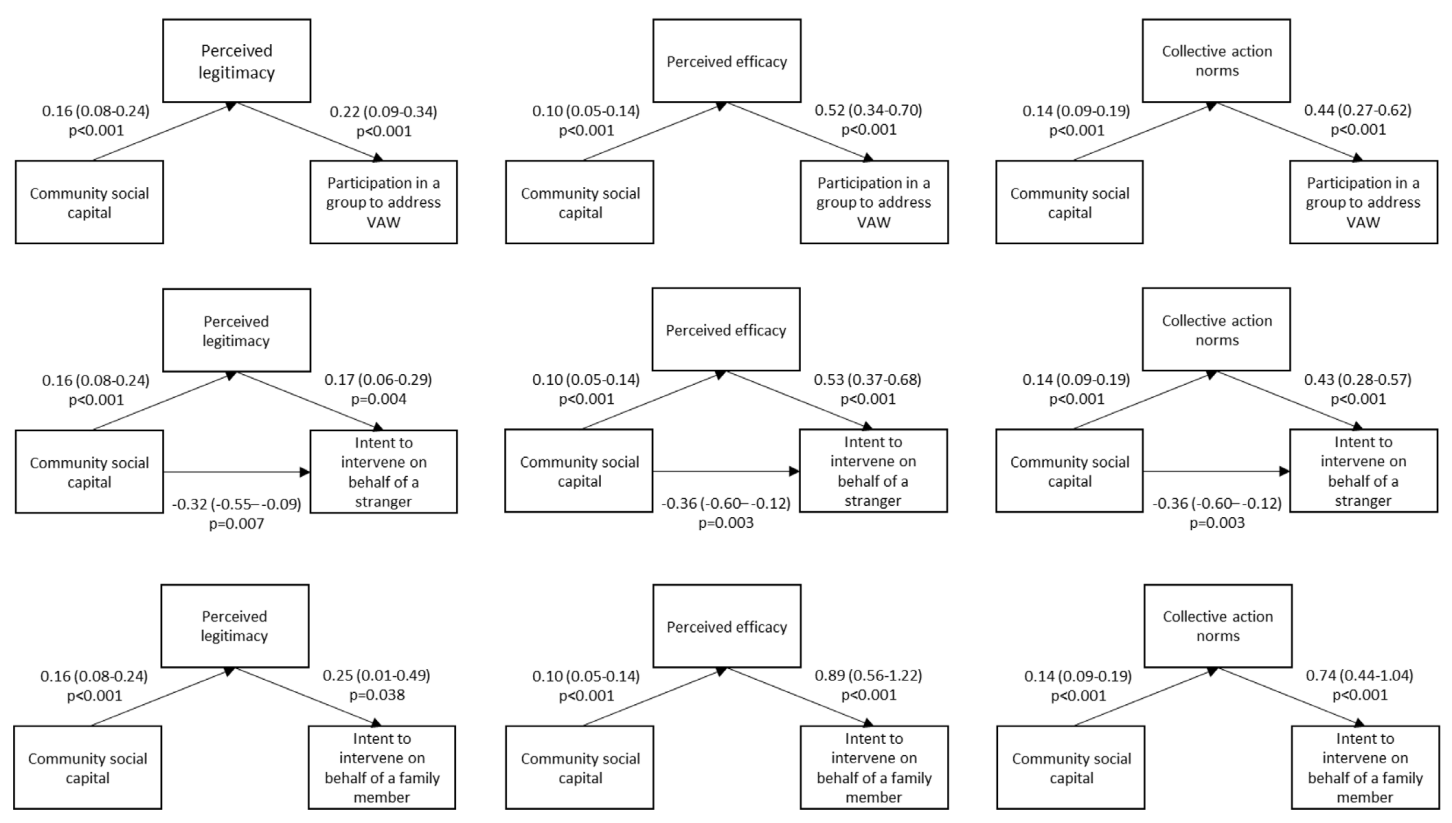
Generalised structural equation models relating social capital and psychological drivers to participation in collective action. N=2,611. Only statistically significant paths (p<0.05) are shown. Regression coefficients are reported in the format of “estimate (95% confidence interval), p-value”. Regression coefficients for paths from predictor variables to behavioural outcomes show the increase in log-odds for one standard deviation increase in the predictor. VAW, violence against women.

We found good evidence that perceived legitimacy, perceived efficacy, and collective action norms related to outcomes, even after adjusting for community social capital ( Figure 3). For each standard deviation increase in perceived legitimacy, odds of participating in a group to address VAW increased 24% (p=0.001, 95% CI 10–41%). For perceived efficacy, odds increased 68% (p<0.001, 40–102%). For collective action norms, odds increased 55% (p<0.001, 30–85%). All three constructs were associated with intent to intervene in case of VAW (p<0.05), with stronger associations for intervening on behalf of a close friend or family member (29–144% increase in odds) than on behalf of a stranger (19–74%).

Social capital was itself positively associated with perceived legitimacy, perceived efficacy, and collective action norms (p<0.001). There was insufficient evidence that social capital was directly associated with participation in groups to address VAW and intent to intervene on behalf of a family member (p>0.05) after adjusting for mediators. Social capital itself was directly negatively associated with intent to intervene on behalf of a stranger, showing a 27–30% reduction in odds of intervening with one standard deviation increase in social capital. However, the indirect effect of social capital on outcomes through perceived legitimacy, perceived efficacy, and collective action norms was positive in all cases ( Table 7). Overall, these results suggest that our three constructs did not simply predict outcomes due to their association with social capital.

**Table 7.** Indirect (mediated) effect of social capital on behavioural outcomes.

Exposure	Mediator	Outcome	OR	p-value	95% CI
Community level social capital	Perceived legitimacy	Participation in a group to address VAW	1.03	0.016	1.01 to 1.06
		Intent to intervene on behalf of a stranger	1.03	0.020	1.00 to 1.05
		Intent to intervene on behalf of a family member	1.04	0.094	0.99 to 1.09
	Perceived efficacy	Participation in a group to address VAW	1.05	0.001	1.02 to 1.08
		Intent to intervene on behalf of a stranger	1.05	<0.001	1.03 to 1.08
		Intent to intervene on behalf of a family member	1.09	0.001	1.04 to 1.14
	Collective action norms	Participation in a group to address VAW	1.06	0.001	1.02 to 1.10
		Intent to intervene on behalf of a stranger	1.06	<0.001	1.03 to 1.10
		Intent to intervene on behalf of a family member	1.11	<0.001	1.05 to 1.18

Table 8 shows associations between individual sub-constructs and our three outcomes. Point estimates showed positive associations with action to address VAW for all sub-constructs except acceptability of male power and control, which showed a negative association; this was consistent with *a priori* theoretical expectations. We found strong evidence that all eight sub-constructs were associated with participation in groups to address VAW (p<0.005). For all sub-constructs except two, we found evidence for an association with intent to intervene in VAW on behalf of a stranger (p<0.005) and a family member (p<0.05). However, we found no evidence for perceived efficacy of specific interventions being associated with intent to intervene in VAW on behalf of a stranger (p=0.102) and for general concern over VAW being associated with intent to intervene in VAW on behalf of either a stranger (p=0.108) or a family member (p=0.334). Overall, this suggests the predictive value of our main three constructs, perceived legitimacy, perceived efficacy, and collective action norms, does not simply derive from a single sub-construct.

**Table 8.** Associations between sub-constructs and action to address violence against women (VAW). N=2,611. All regression analyses have been adjusted for cluster-level social capital. Odds ratios show the increase in odds for one standard deviation increase in the predictor.

Outcome: Participation in groups to address VAW				
Main construct	Sub-construct	OR	p-value	95% CI
Perceived legitimacy	General concern over VAW	1.22	0.003	1.07-1.38
	Acceptability of male power and control	0.82	0.001	0.72-0.92
	Acceptability of intervening in violence	1.24	<0.001	1.10-1.39
Perceived efficacy	Efficacy to achieve specific outcomes	1.37	<0.001	1.20-1.57
	Efficacy of specific interventions	1.30	<0.001	1.17-1.44
	Personal efficacy	1.41	<0.001	1.25-1.60
Collective action norms	Perceived community norms	1.28	<0.001	1.13-1.45
	Perceived family norms	1.32	<0.001	1.16-1.50
Outcome: Intent to intervene in violence against a stranger				
Main construct	Sub-construct	OR	p-value	95% CI
Perceived legitimacy	General concern over VAW	1.14	0.108	0.97-1.34
	Acceptability of male power and control	0.84	0.002	0.75-0.94
	Acceptability of intervening in violence	1.19	0.003	1.06-1.34
Perceived efficacy	Efficacy to achieve specific outcomes	1.40	<0.001	1.27-1.55
	Efficacy of specific interventions	1.10	0.102	0.98-1.23
	Personal efficacy	1.55	<0.001	1.38-1.74
Collective action norms	Perceived community norms	1.31	<0.001	1.18-1.45
	Perceived family norms	1.24	<0.001	1.12-1.37
Outcome: Intent to intervene in violence against a family member				
Main construct	Sub-construct	OR	p-value	95% CI
Perceived legitimacy	General concern over VAW	1.16	0.334	0.86-1.57
	Acceptability of male power and control	0.78	0.042	0.62-0.99
	Acceptability of intervening in violence	1.27	0.045	1.00-1.60
Perceived efficacy	Efficacy to achieve specific outcomes	1.51	<0.001	1.26-1.83
	Efficacy of specific interventions	1.46	0.001	1.18-1.80
	Personal efficacy	1.91	<0.001	1.56-2.35
Collective action norms	Perceived community norms	1.61	<0.001	1.30-2.00
	Perceived family norms	1.56	<0.001	1.27-1.92

## Discussion

To our knowledge, this is the first study to operationalize a measure of the psychological drivers of participation in collective action to address VAW in a low- and middle-income country context. Previous studies of participation in activism against VAW have addressed demographic correlates, but have not measured psychological drivers ^51^. We developed our tool on the basis of a literature review of theories of collective action in social psychology, economics, and political science ^9^. Testing the tool on household survey data collected in urban informal settlements in Mumbai, we found evidence for good item, construct, and criterion validity. Generalised structural equation models showed that our main three hypothesized constructs predicted both intent to intervene in cases of VAW and participation in groups to address VAW, as did almost all of their sub-constructs. Overall, we believe there is sufficient evidence to assert that our scale can provide useful insight into the drivers of collective action to address VAW in our context.

Confirmatory factor analysis revealed an adequate fit of a hierarchical factor structure, in which individual items loaded on first-order factors which themselves loaded on second-order factors representing our three main constructs. However, the sub-construct ‘concern for VAW’ loaded weakly on parent construct perceived legitimacy, had a low internal consistency (ordinal α = 0.662) and was not associated with intent to intervene in case of VAW (p>0.1). It may be that this sub-construct was poorly captured by generic questions on the prevalence and severity of VAW in the respondent’s community. It may also be that abstract concerns over VAW bear little relationship to actual willingness to take action in concrete situations. Social movement researchers have long posited that at any given moment in time there are simply too many different potential causes for an individual to care about for the mere concern with an issue to trigger action ^52, 53^. Future versions of this scale might benefit from measuring alternatives to ‘concern with VAW’.

Our analyses found perceived efficacy and collective action norms were more strongly associated with participation in collective action to address VAW than perceived legitimacy. We emphasize that the primary purpose of this paper was to validate a new measure of possible psychological drivers of collective action, rather provide causal evidence for their role in stimulating action to address VAW. Causality cannot be assumed from our associational analyses due to risks of confounding and reverse causality. Nonetheless, our results provide clues that community mobilisers might benefit from expanding beyond a pure focus on persuading residents of the wrongness of VAW towards engaging with their efficacy and normative beliefs. We also found larger impacts on intent to intervene on behalf of family compared to strangers, indicating it is easier for community mobilisers to encourage action on behalf of family members compared to strangers. In a context in which extended family members often act as perpetrators of violence rather than supporters of victims ^54^, violence prevention programmes might need to emphasise action to support non-family members rather than provide generic calls to action. These findings show the utility of our scale, although further research is required to fully disentangle these complex relationships.

We found no evidence for social capital being positively associated with participation in collective action after adjusting for psychological drivers. Past evidence paints a mixed picture of the role of general social capital in preventing domestic violence, as studies have found it variously beneficial ^55^, harmful ^56^, or neutral ^57^. Although community-level social capital may increase access to support networks for women, it may also empower male community members to police women’s use of such networks ^58^. Social norms disapproving of violence may also be required to translate social capital into action: a trial of a violence prevention programme in Uganda found that social capital was only associated with bystander intervention in intervention areas, not control areas ^51^. We even found that social capital was negatively associated with intent to intervene in case of VAW against a stranger. This echoes literature on the ‘dark side of social capital’ ^59^, which suggests that tightly connected social networks can be detrimental to the health of perceived outsiders by excluding them from the support of network insiders. However, further research is required to unpack the relationship between social capital and VAW.

Surprisingly, we found little evidence for social desirability bias as most items showed little difference in agreement rates between self-administered and face-to-face conditions. Two items that showed more than a five percentage point difference concerned the views of family and neighbours: “your family members consider activities to stop violence against women opposed to their own values” and “you would be embarrassed to say in public that you work to prevent VAW.” As we did not conduct our interviews in private, these differences may reflect respondents feeling better able to voice their opinion when hiding it from their neighbours and family members, rather than from the interviewer. Such biases could be overcome in future surveys by ensuring privacy for the respondent. The third item concerning the effectiveness of sit-ins, strikes, and blockades in stopping VAW might have been interpreted as expressing support for such strategies. Such support might have been controversial to express given the long history of violent clashes between police and residents over forced demolitions of people’s homes in Mumbai’s informal settlements ^60^.

It is possible that the lack of difference between self- and interviewer-administered formats stemmed from respondents feeling insufficiently reassured by the self-administered interview to voice their true opinions. Some respondents may have had difficulty navigating the mobile phone technology on their own, as the proportion of “don’t know” answers increased in the self-administered condition.. However, this may also reflect respondents being more comfortable voicing genuine ignorance in self-administered interviews than face-to-face interviews. There is no perfect way of measuring social desirability. Methods involving list randomization, randomized responses, or bogus pipelines ^61^ are too burdensome for respondents to work in low-literacy, large-N survey settings. Scales for measuring social desirability ^62^ require a leap of faith that biases exhibited on generic trait scales carry over into response patterns for the target construct. Our own manipulation reassured respondents enough to cause an 12-point shift in agreement rates for one item, while the direction of change in other items was generally consistent with respondents feeling free to express less positive attitudes about violence prevention in the self-administered condition. To the best of our knowledge using feasible methods of measuring social desirability bias, we do not have reason to suspect strong hidden bias.

Nonetheless, our scale has limitations. We tried to measure social identity *,* which refers to the extent to which community members feel a sense of shared group membership with others in their reference group ^22^. We wanted to measure politicized collective identity as being an ‘activist’ ^63^, since the category of ‘women’ as a whole had been criticized for being too large, vague, and internally divided to constitute an effective identity for feminist activism ^64^. However, prior measures of activist identities for gender equality have asked respondents to self-identify as ‘feminists’ ^65^, a term that was poorly understood in our setting. Items asking respondents if respondents thought themselves ‘similar to’ ^66^ activists trying to stop VAW were taken too literally and elicited the response that it would be impossible to know for sure as they had never met such people in person. Similarly, questions about whether respondents ‘had a bond with’, ‘felt connected to’, or ‘felt strong ties with’ ^67^ such activists elicited the response that they had never met such people, so how could they have ties with them? Asking people if they considered themselves part of the ‘women’s movement’ was interpreted to mean participation in protest, as the term ‘movement’ ( *andolan*) primarily signified mass protest. Questions asking respondents if they saw themselves as ‘the kind of person’ ^68^ who would take action against VAW ended up simply reflecting whether they in fact had taken such action. In the end, we decided not to measure this construct, but we cannot rule out the possibility that future researchers might discover creative ways of capturing this construct.

Our scale also relied on asking people whether they were willing to engage in activism to address ‘violence against women’ ( *mahila ke khilaaf hinsa*) or ‘domestic violence’ ( *gharelu hinsa*). The World Health Organization and the Demographic Health Surveys recommend avoiding the term ‘violence’ wherever possible, as there is considerable individual variation in respondent interpretation of the term, including a tendency to consider only extreme practices (e.g. beating or choking) forms of violence, whilst ignoring ‘milder’ practices (e.g. slapping) ^69, 70^. However, in a survey setting, it would be unreasonably unwieldy to ask separate questions for attitudes to action against slapping, attitudes to action against kicking, attitudes to action against forced sex, etc. Global health researchers thus universally invoke the generic term ‘violence’ in questions on participation in action to address VAW ^51, 71^ , as do researchers on bystander intervention ^72^. However, this practice does cause confusion: respondents sometimes asked during interviews what the word ‘violence’ meant, which required interviewers to clarify. We piloted alternatives for the word ‘violence’, but these created even more confusion – ‘forcing/coercing’ someone ( *zabardasti karna*) has the unfortunate alternate sense of ‘insisting on something’, while the word ‘force’/ bal does not have an intrinsic negative valence like the word ‘violence’ and may even have positive connotations of ‘strength’ and ‘power’. In future research, there may be merit in exploring more blunt phrases such as ‘action to stop husbands beating their wives’ as proxies for ‘action to address violence’. During piloting, respondents said it was hard to distinguish collective action against different forms of VAW, as physical, sexual, and emotional violence tended to occur together for survivors of violence.

Finally, our study was limited by missing data, as 30% of respondents lacked data for at least one indicator. However, at most 8% of respondents did not know the answer to any single indicator. As stated earlier, we used complete case analysis for item validity on an item-by-item basis, while we used imputation to create an index of items to assess criterion validity. As respondents with missing data were more likely to be poorly educated, this may have upwardly biased assessments of scale validity.

## Conclusion

We present a new scale for measuring the psychological drivers of collective action to prevent VAW, developed in the context of a community mobilisation programme in urban India. Our scale may offer fresh clues to modifiable beliefs and attitudes that global health interventions can address to maximally inspire activism. Discovering clues is highly relevant for a policy landscape in which participatory approaches to gender equality and health are rapidly gaining momentum ^73^. We invite researchers and practitioners to adapt and test our scale in their own contexts in order to advance our knowledge of pathways to activism.

## Data availability

### Underlying data

Open Science Framework: Measuring the psychological drivers of participation in collective action to address violence against women in Mumbai, India. v2.0 10.17605/OSF.IO/W2VSY ^29^.

This project contains the following underlying data:

- Data Validation Study.csv (cleaned data for study in CSV format)
- Codebook For Data.csv (codebook for the above data file)

### Extended data

Open Science Framework: Measuring the psychological drivers of participation in collective action to address violence against women in Mumbai, India. v2.0 10.17605/OSF.IO/W2VSY ^29^.

This project contains the following extended data:

- Supplementary Table 1.docx (survey items grouped by construct)
- Supplementary Table 2.docx (comparison of respondents with and without missing data)
- Supplementary Table 3.docx (levels of social capital reported by respondents)

Data are available under the terms of the Creative Commons Attribution 4.0 International license (CC-BY 4.0).
