# Supplementary material for: Measuring the psychological drivers of participation in collective action to address violence against women in Mumbai, India
Source: Wellcome Open Res. 2020 Jun 29;5:22. Originally published 2020 Feb 10. [Version 2] doi: 10.12688/wellcomeopenres.15707.2 (PMC7281673; doi:10.12688/wellcomeopenres.15707.2)
Supplement: Peer review history

# Peer review: Reviewer response for version 2

**DOI:** 10.21956/wellcomeopenres.17648.r39284

**Published:** 2020-08-04

**Authors:** Nafisa Halim

**Affiliations:** 1 Department of Global Health, Boston University School of Public Health, Boston, MA, USA

Thank you for clarifying! I would ask the authors to make a slight amendment here. First question: do they agree with (1) legitimacy of actors/organizations is an important construct for understanding the outcome; however, it can't be measured using their data; or (2) legitimacy is not important and doesn’t need to be measured. If they agree with (1), I suggest including lack of measurement as a data limitation. If they agree with (2), the authors should add a sentence summarizing their argument with citations (citations are key since I could not tell if their arguments were evidence based or opinions. For example, when the authors say “However, that is rarely how community mobilisation interventions operate, as they encourage behaviour change through participation rather than coercion,” Yes, of course, ideally this is how community mobilization interventions should work! The question is about implementation fidelity. Please cite some process evaluation studies on community mobilizations assessing implementation fidelity.

Is the work clearly and accurately presented and does it cite the current literature?

Yes

If applicable, is the statistical analysis and its interpretation appropriate?

Yes

Are all the source data underlying the results available to ensure full reproducibility?

Yes

Is the study design appropriate and is the work technically sound?

Yes

Are the conclusions drawn adequately supported by the results?

Yes

Are sufficient details of methods and analysis provided to allow replication by others?

Yes

Reviewer Expertise:

I am a violence prevention researcher.

I confirm that I have read this submission and believe that I have an appropriate level of expertise to confirm that it is of an acceptable scientific standard.

# Peer review: Reviewer response for version 2

**DOI:** 10.21956/wellcomeopenres.17648.r39283

**Published:** 2020-07-20

**Authors:** Kathryn M. Yount

**Affiliations:** 1 Hubert Department of Global Health ad Department of Sociology, Emory University, Atlanta, GA, USA

Is the work clearly and accurately presented and does it cite the current literature?

Yes

If applicable, is the statistical analysis and its interpretation appropriate?

Partly

Are all the source data underlying the results available to ensure full reproducibility?

Yes

Is the study design appropriate and is the work technically sound?

Partly

Are the conclusions drawn adequately supported by the results?

Partly

Are sufficient details of methods and analysis provided to allow replication by others?

Partly

Reviewer Expertise:

Measurement; violence against women prevention.

I confirm that I have read this submission and believe that I have an appropriate level of expertise to confirm that it is of an acceptable scientific standard.

# Peer review: Reviewer response for version 1

**DOI:** 10.21956/wellcomeopenres.17216.r38637

**Published:** 2020-06-22

**Authors:** Nafisa Halim

**Affiliations:** 1 Department of Global Health, Boston University School of Public Health, Boston, MA, USA

Thank you for the opportunity to review this interesting paper by Gram and colleagues. The paper attempts to fill out a critical gap in the violence prevention literature —the psychosocial determinants of community voluntary participation in violence prevention efforts. The study was conducted in an LMIC, which is novel as a study context. The authors used a reasonable theoretical framework; original data; an adequately sized sample; appropriate analytical strategies. I’ll keep my comments to a selected few.

1. It seems to be that the authors relied on a value expectancy model like health belief model in formulating their hypothesis of the factors helping community members to If this correct? If yes, the authors might want to be explicit about their use of a value expectancy model, and make a case for why a value expectancy model would apply to frame factors affecting motivation among the study population (i.e., namely vulnerable, low-literacy populations living in informal settlements in Mumbai). If not, the authors might want to use a framework to inform their hypothesis and use of three constructs.
2. Related to # 1, I could also argue that community members may be motivated through beliefs in the legitimacy of the external agents. Community members may consider external agents as legitimate authority figures to carry out violence prevention efforts if the agents: provide them with material incentives: have social ties with the political elites, or there are normative appeals for the external agents in the community (i.e., the agents represent a virtue [e.g., education, nationality, etc.] deemed desirable or valued in the community).
3. The authors define *Perceived legitimacy as “*This construct refers to the extent to which action against VAW is seen as a legitimate endeavour.” This definition seems tautological to me (defining legitimacy with legitimacy), unless I am missing something obvious here. Also, lacking an adequate conceptualization, the basis of dividing perceived autonomy into three sub-constructs appears to be a bit ad hoc. Also, I would think that “General concern about VAW” would be a predictor of Perceived Legitimacy, and not a dimension or a sub-construct. I think I am struggling with perceived legitimacy as a construct and its relation with the sub-constructs.
4. The authors might want to disaggregate their main analysis by gender. Since the practice world seems to have transitioned from targeting women to targeting men to targeting both men and women for violence prevention interventions, understanding whether and to what extent men and women are different in intention to participate in community activism might be helpful for programmatic purposes.
5. I was curious as to why the authors did not control for the demographic correlates of participation in collective action to address VAW. If the objective of this study is to inform the practice world about the ways of mobilizing masses, understanding the contribution of psychometric factors relative to that of demographic factors would have been most useful, I think. Since the authors are likely to have access to the data on demographic correlates, why not rerun the model by adjusting for demographic correlates.
6. Perhaps I missed it, I wonder about the goodness of fit statistics for the models in Table 6.
7. I don’t know where #7 fits, or if this fits for this analysis at all, but I could not help but wondering about each of perceived legitimacy, perceived efficacy, and perceived norms being context-dependent (and therefore not a random occurrence!). We tend to formulate a perception or an opinion by applying a deliberate system of thinking (i.e., rational choice) or an automatic system of thinking (i.e., mental models, learned behavior). Given the study population, who allegedly may have limited access to information or knowledge about VAW, its likely their perceptions have been informed by community perceptions or by the perception of individuals of influence in the community. Do the authors agree with this argument? If yes, does it make sense to fit multilevel models as opposed to individual level models in Table 6. Also, I think the authors have done it, but at a minimum the error terms should be adjusted for community level clustering.
8. Was this by design that the authors sampled 500 households from 54 informal settlement clusters. How many households are there per cluster on the average? What was the sampling strategy—simple probably or proportional probably sampling? If simple, there are potentially 9-10 households from some clusters. If yes, calculation of standard errors needs to be adjusted for unequal number of households per cluster or potentially small number of households in some clusters. Minor point, but perhaps worth exploring, especially since the authors have adjusted for social capital levels at the cluster level.

Is the work clearly and accurately presented and does it cite the current literature?

Yes

If applicable, is the statistical analysis and its interpretation appropriate?

Yes

Are all the source data underlying the results available to ensure full reproducibility?

Yes

Is the study design appropriate and is the work technically sound?

Yes

Are the conclusions drawn adequately supported by the results?

Yes

Are sufficient details of methods and analysis provided to allow replication by others?

Yes

Reviewer Expertise:

I am a violence prevention researcher.

I confirm that I have read this submission and believe that I have an appropriate level of expertise to confirm that it is of an acceptable scientific standard, however I have significant reservations, as outlined above.

# Response

**Published:** 2020-06-23

**Authors:** Lu Gram

**Affiliations:** University College London, UK

Thank you for your thorough critique and interesting, novel insights. We have revised the manuscript in accordance with your suggestions, wherever possible.

*1. It seems to be that the authors relied on a value expectancy model like health belief model in formulating their hypothesis of the factors helping community members to If this correct? If yes, the authors might want to be explicit about their use of a value expectancy model, and make a case for why a value expectancy model would apply to frame factors affecting motivation among the study population (i.e., namely vulnerable, low-literacy populations living in informal settlements in Mumbai). If not, the authors might want to use a framework to inform their hypothesis and use of three constructs.*

This is an interesting comment. We appreciate the similarity between our conceptualisation and frameworks such as the Health Belief Model or the Theory of Planned Behaviour. The literature on collective action is vast and each of the constructs, legitimacy, efficacy, and norms are independently associated with a sprawling body of theoretical literature [3, 7]. For example, an enduring debate exists over whether such constructs represent conscious deliberation, heuristics, periphrastic shorthands for causes of behaviour, equilibrium outcomes of individual learning, equilibrium outcomes of cultural evolution, etc. [8-10]. Given restrictions on the level of detail we can realistically obtain through surveys in LMIC settings, we have abstracted away from these considerations in our framework. These distinctions may also matter ultimately less for programmatic purposes: if perceived legitimacy, efficacy and norms affect participation in collective action and intervention planners find low levels of these at baseline, it makes sense for them to address it regardless of the precise interpretation of this relationship in terms of the above theories. We have referenced a past paper discussing overarching theory and are hesitant to over-complicate the current paper by adding further theory into the introduction [1].

*2. Related to # 1, I could also argue that community members may be motivated through beliefs in the legitimacy of the external agents. Community members may consider external agents as legitimate authority figures to carry out violence prevention efforts if the agents: provide them with material incentives: have social ties with the political elites, or there are normative appeals for the external agents in the community (i.e., the agents represent a virtue [e.g., education, nationality, etc.] deemed desirable or valued in the community).*

Thank you for this comment. Our measure mainly seeks to capture the legitimacy of action against VAW as opposed to the legitimacy of external organisations working in this field. Poor, vulnerable women face overwhelming pressure to say to local service providers, NGOs and government agents that they are legitimate actors. As such, perceived legitimacy of organisations is unlikely to be captured accurately through a survey, even were we to use self-administered surveys to ask about this.

Perceived legitimacy of organisations also most likely increases participation in collective action *through *its effect on perceived legitimacy of action, perceived efficacy of action, and collective action norms. It is of course possible that community members will organise collective action to prevent VAW simply because they are told to do so by a reputable organisation, even if they personally believe such action is unjust, ineffective and embarrassing to do.

However, that is rarely how community mobilisation interventions operate, as they encourage behaviour change through participation rather than coercion. In the field of VAW prevention in particular, there are strong ethical objections to using material or political incentives to promote action against VAW – rewarding community members for identifying, referring or supporting survivors of violence in monetary terms or through political access would create perverse incentives for community members to ‘find’ more cases of violence.

For all the above reasons, we opted not to measure legitimacy of organisations in our scale.

*3. The authors define Perceived legitimacy as “This construct refers to the extent to which action against VAW is seen as a legitimate endeavour.” This definition seems tautological to me (defining legitimacy with legitimacy), unless I am missing something obvious here. Also, lacking an adequate conceptualization, the basis of dividing perceived autonomy into three sub-constructs appears to be a bit ad hoc. Also, I would think that “General concern about VAW” would be a predictor of Perceived Legitimacy, and not a dimension or a sub-construct. I think I am struggling with perceived legitimacy as a construct and its relation with the sub-constructs.*

We have defined perceived legitimacy similarly to how we defined perceived efficacy as the extent to which action was seen as effective. We conceptualised as primarily related to our statement about community members being motivated (or demotivated) by the intrinsic rightness (or wrongness) of participation in collective action:

To overcome such dilemmas, community members may be motivated through beliefs in the intrinsic rightness of participation in activism ^13^ …

(from Section: Theoretical Framework)

As such, participation in collective action to address VAW can be seen as more justified, the more community members are concerned about VAW, believe men have no right to control and dominate women, and feel interventions in cases of VAW do not violate privacy norms. We have now revised the paragraph to clarify this.

*Perceived legitimacy.* This construct refers to the extent to which action against VAW is seen as justified. It aligns with a number of theories positing that perceived grievance, injustice, or deprivation ^13^ motivate collective action for social change, while perceived justification for the status quo demotivates collective action ^16^ . We divided the construct into three sub-constructs referring to respondent concern about VAW in general, acceptance of male power and control in the household, and beliefs about the acceptability of intervening in cases of VAW. These three sub-constructs are thought of as contributing to respondent feelings regarding the intrinsic rightness (or wrongness) of action against VAW.

(from Section: Theoretical Framework)

The division into three sub-constructs was based on feminist literature, which ascribes a major role to norms of privacy and male domination over women in perpetuating VAW [11]. The construct, “general concern about VAW,” was informed by past conceptual models of community mobilisation which have included a dimension of “shared concern” with a particular issue [12]. For policy purposes, we thought it was relatively clear that differences in the influence of privacy norms, beliefs about male domination and control, and general concern for VAW had distinct programmatic implications.

However, we agree that further theoretical work may clarify the structure of our ‘legitimacy’ construct. We wanted to avoid an extended discussion on its exact meaning given the heterogeneous literature associated with it. Longer and precise definitions of unobservable constructs might be less ambiguous, but complete clarity can never be attained [13]. Highly specific questions about big concepts may produce more precise, but less valid, measurement, as the questions become over-specific [14]. As such, we have not significantly expanded our discussion of the concept in this paper.

*The authors might want to disaggregate their main analysis by gender. Since the practice world seems to have transitioned from targeting women to targeting men to targeting both men and women for violence prevention interventions, understanding whether and to what extent men and women are different in intention to participate in community activism might be helpful for programmatic purposes.*

We agree that it is important to disaggregate by gender for programmatic purposes. As stated in the discussion, we wanted the primary focus of the paper to be on measurement and look into substantive issues in forthcoming papers. However, we agree that it may be worthwhile to look at gender differences in the response to our measure. We have divided this task into three sub-tasks: looking at gender differences in the make-up of factor structure, looking at gender differences in the response to social desirability items, and looking at gender differences in convergent and criterion validity.

Gender differences in factor structure:

We conducted measurement invariance tests across gender, education, caste and marital status and found little evidence for differences in model fit by these groups. The table is displayed in the Results section of the revised manuscript. We added the following text:

We did not find strong evidence against measurement invariance (Table 7). Comparing fit across models accounting for gender, education, caste, and marital status, both WRMR and TLI decreased slightly as constraints on factor loadings were lifted, while RMSEA stayed relatively constant. The decrease in WRMR indicates better fit for models allowing for heterogeneity across groups, but the decrease in TLI indicates a worse fit, possibly due to lower parsimony in the unconstrained models. Given these results, we did not see a need for separate measurement models by gender or demographic group.

(from Section: Results – construct validity)

Gender differences in social desirability:

We have also tested for interactions with these variables in measuring social desirability bias. Here, we find little evidence that female respondents were more strongly affected by social desirability bias than male respondents. The only item for which we found a significant interaction showed men displaying greater social desirability effects than women:

With respect to gender differences in social desirability, we found no evidence for an interaction with gender in all indicators (p>0.05) except for the item “Your family members approve of you joining activities to stop violence against women” (p=0.008), where the self-administered interview had a clear effect on men’s odds of agreeing (OR 0.51, p=0.037, 95% CI 0.27-0.97), but not women’s odds (OR 1.36, p=0.20, 95% 0.85-2.19).

(from Section: Results – item validity)

Gender differences in associations between psychological constructs and behavioural outcomes:

As mentioned above, we wanted the primary focus of the paper to be on measurement and look into substantive issues in forthcoming papers. While it is possible, even likely, that men and women will show different associations between different psychological drivers and participation in collective action, we do not have strong enough evidence to make a priori hypotheses which could inform us if the patterns observed in the data indicate high or low validity for our scale. As such, we think this issue is better explored in a separate paper looking at unpacking these associations in more depth, which we are currently preparing.

*I was curious as to why the authors did not control for the demographic correlates of participation in collective action to address VAW. If the objective of this study is to inform the practice world about the ways of mobilizing masses, understanding the contribution of psychometric factors relative to that of demographic factors would have been most useful, I think. Since the authors are likely to have access to the data on demographic correlates, why not rerun the model by adjusting for demographic correlates.*

The results do not change when adjusted for demographic correlates. As stated above, we wanted the primary focus of the paper to be on measurement and disentangle issues of reverse causality, confounding, and effect modification in forthcoming papers. It is conventional in measurement theory to use simple correlations to check for convergent validity – just like factor analysis is carried out on crude correlation matrices – so we have kept adjustments to the bare minimum [15-17]. We have briefly mentioned substantive implications of our results to illustrate how our scale might be used in practice. We have now further clarified this in the main text:

These findings show the utility of our scale, although further research is required to fully disentangle these complex relationships.

(from Section: Discussion)

*Perhaps I missed it, I wonder about the goodness of fit statistics for the models in Table 6.*

These are simple logistic regression models, so comparable goodness of fit statistics as those displayed in Table 4 are not available for them. Goodness of fit statistics for logistic regression models such as Hosmer-Lemeshow χ ^2^ or Tukey’s link test are sensitive to omitted variable bias, which is not relevant, when we are primarily using the models to establish simple correlations to assess convergent validity.

*I don’t know where #7 fits, or if this fits for this analysis at all, but I could not help but wondering about each of perceived legitimacy, perceived efficacy, and perceived norms being context-dependent (and therefore not a random occurrence!). We tend to formulate a perception or an opinion by applying a deliberate system of thinking (i.e., rational choice) or an automatic system of thinking (i.e., mental models, learned behavior). Given the study population, who allegedly may have limited access to information or knowledge about VAW, its likely their perceptions have been informed by community perceptions or by the perception of individuals of influence in the community. Do the authors agree with this argument? If yes, does it make sense to fit multilevel models as opposed to individual level models in Table 6.*

This is an interesting idea. As mentioned in our answer to Point 1, there is a large and lively debate as to the extent to which human preferences, beliefs and norms have come about through deliberation, imitation, learning, cultural evolution, etc. For the purpose of this paper, we are simply investigating whether we can measure perceived legitimacy, perceived efficacy, and perceived norms at an individual level. Further research is required to establish the precise ways in which individual perceptions interact at a community level. We are not sure we can assume one mechanism rather than another at this stage. 56% of women and 46% of men do not recognise most people in their neighbourhood (Supplementary Table 3), so there may not necessarily be strong peer effects operating at a community level.

*Also, I think the authors have done it, but at a minimum the error terms should be adjusted for community level clustering.*

Indeed, the error terms are already adjusted for community level clustering.

*Was this by design that the authors sampled 500 households from 54 informal settlement clusters. How many households are there per cluster on the average? What was the sampling strategy—simple probably or proportional probably sampling? If simple, there are potentially 9-10 households from some clusters. If yes, calculation of standard errors needs to be adjusted for unequal number of households per cluster or potentially small number of households in some clusters. Minor point, but perhaps worth exploring, especially since the authors have adjusted for social capital levels at the cluster level.*

The data were collected in a survey conducted before our community intervention began in a cluster randomised controlled trial [18]. The trial includes 54 clusters of ~500 households each, identified within much larger agglomerated informal settlements. We took a systematic random sampling approach similar to other surveys: clusters were selected at random and households within them visited systematically until 25 women and 25 men had completed questionnaires in each cluster. The clusters were of equal size and each yielded ~50 questionnaires. We have now clarified this:

Between December 2017 and December 2019, we carried out a baseline survey of community attitudes to VAW in households across 54 informal settlement clusters. Each cluster contained about 500 households. Clusters were in four large urban informal settlements, chosen for their vulnerability, low risk of rehabilitation, low coverage by organisations working to address VAW, and low proportion of rental tenancies. From a random starting point in each cluster, 16 investigators selected 25 women and 25 men aged 18 to 65 years – a single interviewee per household – by visiting households sequentially. We thus obtained approximately 50 interviews per cluster. Participants were enrolled in person. Inclusion criteria were that respondents should fall into these age groups and should provide signed consent.

(from Section: Data Collection)

**References**

1.         Gram, L., N. Daruwalla, and D. Osrin, *Understanding participation dilemmas in community mobilisation: can collective action theory help?* J Epidemiol Community Health, 2019. **73**(1): p. 90-96.

2.         Gram, L., et al., *Promoting women and children's health through community-based groups in low- and middle-income countries: a mixed methods systematic review of mechanisms, enablers and barriers.* BMJ Global Health, 2019. **4**: p. e001972.

3.         Van Zomeren, M., T. Postmes, and R. Spears, *Toward an integrative social identity model of collective action: a quantitative research synthesis of three socio-psychological perspectives.* Psychological Bulletin, 2008. **134**(4): p. 504.

4.         Ostrom, E., *A behavioral approach to the rational choice theory of collective action: Presidential address, American Political Science Association, 1997.* American Political Science Review, 1998. **92**(1): p. 1-22.

5.         Fokkema, M. and S. Greiff, *How performing PCA and CFA on the same data equals trouble*. 2017, Hogrefe Publishing.

6.         Clark, C.J., et al., *Social norms and women's risk of intimate partner violence in Nepal.* Social Science & Medicine, 2018. **202**: p. 162-169.

7.         Ostrom, E., *Collective action and the evolution of social norms.* Journal of Natural Resources Policy Research, 2014. **6**(4): p. 235-252.

8.         Kahneman, D., *Maps of bounded rationality: Psychology for behavioral economics.* The American Economic Review, 2003. **93**(5): p. 1449-1475.

9.         Henrich, J. and R. McElreath, *Dual inheritance theory: the evolution of human cultural capacities and cultural evolution.* Oxford handbook of evolutionary psychology, 2007: p. 555-570.

10.       Andreoni, J. and J.H. Miller, *Analyzing choice with revealed preference: is altruism rational?* Handbook of experimental economics results, 2008. **1**: p. 481-487.

11.       Heise, L.L., *Violence against women an integrated, ecological framework.* Violence against women, 1998. **4**(3): p. 262-290.

12.       Lippman, S.A., et al., *Conceptualizing community mobilization for HIV prevention: implications for HIV prevention programming in the African context.* PloS one, 2013. **8**(10): p. e78208.

13.       Gram, L., J. Morrison, and J. Skordis-Worrall, *Organising concepts of ‘women’s empowerment’ for measurement: a typology.* Social Indicators Research, 2018: p. 1-28.

14.       King, G., et al., *Enhancing the validity and cross-cultural comparability of measurement in survey research.* American Political Science Review, 2004. **98**(1): p. 191-207.

15.       Gram, L., et al., *Validating an Agency-Based Tool for Measuring Women's Empowerment in a Complex Public Health Trial in Rural Nepal.* Journal of Human Development and Capabilities, 2016: p. 1-29.

16.       Abbasi, M., et al., *A cross-sectional study examining convergent validity of a frailty index based on electronic medical records in a Canadian primary care program.* BMC geriatrics, 2019. **19**(1): p. 109.

17.       Ljótsson, B., et al., *Discriminant and convergent validity of the GSRS-IBS symptom severity measure for irritable bowel syndrome: A population study.* United European Gastroenterology Journal, 2020. **8**(3): p. 284-292.

18.       Daruwalla, N., et al., *Community interventions to prevent violence against women and girls in informal settlements in Mumbai: the SNEHA-TARA pragmatic cluster randomised controlled trial.* Trials, 2019. **In Press**.

# Peer review: Reviewer response for version 1

**DOI:** 10.21956/wellcomeopenres.17216.r38810

**Published:** 2020-06-08

**Authors:** Shireen Jejeebhoy

**Affiliations:** 1 Aksha Centre for Equity and Wellbeing, Mumbai, India

This article contributes to the growing body of evidence on violence against women in India, and is unique in several ways and goes beyond other studies in several ways. The study, situated in informal settlements in Mumbai, India, focuses on a rarely studied dimension, namely the key factors associated with participation in collective action to address such violence. In doing so, it presents an evidence-informed and thoughtful theoretical framework, and also develops an evidence-informed scale to measure three drivers of collective action, that is, perceived legitimacy, perceived efficacy and collective action norms. And it tests, in various ways, the robustness of this scale and its components - social desirability, by comparing responses using self-administered and face-to-face interviewing techniques; acquiescence bias by comparing positively and negatively worded questions; construct validity; and criterion validity. Authors conclude that their scale is indeed robust, offering “clues to modifiable beliefs and attitudes that global health interventions can address to maximally inspire activism”. Although the methodology appears to me to be sound and well-explained, I do not feel qualified to comment on its rigour and appropriateness. I have a few observations on other issues.

 Objectives are not clear, and a clear statement of objectives is needed. It appears to be both “to develop a new scale measuring drivers of participation in collective action to address VAW” and to better understand the associations of perceived efficacy, collective action norms and perceived legitimacy with participation in collective action. Although there is a considerable discussion of the latter, authors emphasise that the “sole” purpose of the paper is to validate the new measure of drivers. Obviously causality cannot be assessed from a cross-sectional study, but associations certainly can. Authors need to be very clear about what the objectives are, and if one is the “sole” purpose of the paper, justify the need to address the second as well. 

 The study was based on a survey of 2642 respondents residing in 54 informal settlements of the city. The sample was equally divided between men (1307) and women (1311) aged 18-65, and analysis was performed for the entire sample. Would a gender-segmented analysis have provided authors with a more nuanced set of insights? As is well known, India is a patriarchal and gender stratified society, characterised by men exercising a sense of entitlement to control women, and women largely submissive to unbalanced gender norms (see for example, Santhya *et al.* 2013 ^1^; Jejeebhoy *et al.* 2013 ^2^). Domestic violence is widespread, with one-third of married women reporting an experience; it is, moreover, widely justified, with half (52%) of women and two-fifths (42%) of men justifying violence for at least one of a host of reasons, including going out without permission, neglecting the household and disrespecting in-laws (International Institute for Population Sciences (IIPS) and ICF, 2017 ^3^). In this hierarchical setting, it is likely that men and women will respond differently to the items on the scale developed in this study, for example, embarrassment to declare engagement in violence prevention work, or attitudes about whether protests are effective in stopping violence, and differences would emerge in perceived legitimacy, efficacy and social norms, as well as social desirability responses. Even if the sample size does not permit a sub-group analysis, it seems worthwhile to conduct the analysis separately for men and women to observe any key disparities, with regard to both the scale as well as the correlates.

 Authors themselves raise questions about their respondents’ understanding of the term ‘violence against women’ and note that they required to explain the term to respondents in some instances. Given that the whole study focuses on violence against women, this possible ambiguity in understanding of the term is cause for concern. Both the World Health Organisation (World Health Organisation, 2005) ^4^ and the Demographic and Health Surveys (see, for example, Kishor and Johnson, 2004) ^5^ have recognised this ambiguity, and have posed, instead of a single question, a battery of questions relating to different acts of physical violence, as well as sexual and emotional violence. Both research programmes recognise the need to specifically define various acts of violence precisely so that the violence measure is not affected by different understandings of what constitutes violence. In India, many associate the term ‘violence’ with extreme practices such as severe beating that results in injury, choking and so on; a slap is an everyday event that few would consider violence, and few would agree that participation in action to counter such an act is warranted. Authors describe steps taken if the respondent asked for clarification, but it is likely that others who did not request clarification may have had a limited definition of violence. An introduction that defines what is implied by the term and applied to all respondents may have reduced these ambiguities in terminology. While this limitation cannot be addressed, it would be helpful to highlight this limitation at greater length, and speculate about possible effects on the scale or other outcomes.

 Given the limited voice that women and girls exercise in families in India, many personal questions can raise discomfort levels among them. Hence, typically, sections of surveys dealing with these issues are conducted in private, without mothers and mothers-in-law listening in. Without an assurance of privacy, it is very possible that women’s responses would be more likely to adhere to traditional norms and expectations than if privacy is ensured. In this case, while it is true that personal experiences are not elicited, attitudes about the acceptability of intervention and collective action or perceptions about how families would react to their participation in collective action to stop a behaviour that is perceived as so acceptable, may also cause discomfort to young women who are audible to their mothers-in-law, and result in responses that are acceptable to those listening in. While here too, nothing can be done now, it would be helpful to highlight this limitation and offer some ideas about how it might have affected findings.

 Small points. Social capital is discussed, and adjustments made for it, but there is no indication in the paper of the level of social capital in the study sample, it would be good to highlight this or explain why it has been omitted. Also, authors may want to think of a friendlier way of displaying the information provided in Figure 3. 

 Overall, the paper addresses important and poorly understood issues relating to participation in community action. It is perhaps the only study of its kind in India that has measured social desirability and acquiescence biases, has developed a scale to encompass the various drivers of participation, and has shown the extent to which these constructs are associated with outcomes such as participation in groups and intent to intervene in incidents of violence. It is very well-written, if dense. My suggestions for revision are relatively small, and call for a more consistent statement of objectives, a possible gender segmented analysis, and a stronger acknowledgement of study limitations.

Is the work clearly and accurately presented and does it cite the current literature?

Yes

If applicable, is the statistical analysis and its interpretation appropriate?

Yes

Are all the source data underlying the results available to ensure full reproducibility?

Yes

Is the study design appropriate and is the work technically sound?

Partly

Are the conclusions drawn adequately supported by the results?

Yes

Are sufficient details of methods and analysis provided to allow replication by others?

Yes

Reviewer Expertise:

Gender, notably female empowerment, violence against women; adolescent health and development; sexual and reproductive health and rights; India; progamme evaluation.

I confirm that I have read this submission and believe that I have an appropriate level of expertise to confirm that it is of an acceptable scientific standard.

## References

[1] : Growing up in rural India: An exploration into the lives of younger and older adolescents in Madhya Pradesh and Uttar Pradesh.2013; 10.31899/pgy3.1011 10.31899/pgy3.1011

[2] : Gender-based violence: A qualitative exploration of norms, experiences and positive deviance.2013; 10.31899/rh3.1013 10.31899/rh3.1013

[3] : National Family Health Survey (NFHS-4), 2015-16.2017;

[4] : WHO multi-country study on women’s health and domestic violence against women: summary report of initial results on prevalence, health outcomes and women’s responses.2005;

[5] : Profiling Domestic Violence – A Multi-Country Study.2004;

# Response

**Published:** 2020-06-23

**Authors:** Lu Gram

**Affiliations:** University College London, UK

We appreciate you taking your time to provide your valuable insights from your many years of working in this field and in this context. We have implemented your constructive suggestions as much as possible.

*Objectives are not clear, and a clear statement of objectives is needed. It appears to be both “to develop a new scale measuring drivers of participation in collective action to address VAW” and to better understand the associations of perceived efficacy, collective action norms and perceived legitimacy with participation in collective action. Although there is a considerable discussion of the latter, authors emphasise that the “sole” purpose of the paper is to validate the new measure of drivers. Obviously causality cannot be assessed from a cross-sectional study, but associations certainly can. Authors need to be very clear about what the objectives are, and if one is the “sole” purpose of the paper, justify the need to address the second as well.*

Thank you for this. We completely understand your concern. There is a chicken-and-egg problem in building measurement and substantive theory in that we need valid measurements to provide evidence for substantive theory, but substantive theory is also needed to interpret and validate new measures. In a relatively nascent subject like women’s collective action to prevent VAW, we have neither prior measures to compare against nor substantive theory backed by strong evidence. Past scale validation studies running into this problem have combined the two objectives into one [6]. We opted for running checks on convergent and discriminant validity using simple correlations and regressions, while leaving the detailed disentanglement of associations between our measure and outcomes to a follow-up paper (which we are writing). The brief discussion of the implications of different magnitudes of associations with perceived efficacy, legitimacy and norms was merely meant to illustrate how our scale might be useful to policy-makers. To clarify this, we have now replaced “sole” with “primary” and added a sentence to the end of the paragraph:

These findings show the utility of our scale, although further research is required to fully disentangle these complex relationships.

(from Section: Discussion)

*The study was based on a survey of 2642 respondents residing in 54 informal settlements of the city. The sample was equally divided between men (1307) and women (1311) aged 18-65, and analysis was performed for the entire sample. Would a gender-segmented analysis have provided authors with a more nuanced set of insights? As is well known, India is a patriarchal and gender stratified society, characterised by men exercising a sense of entitlement to control women, and women largely submissive to unbalanced gender norms (see for example, Santhya et al. 2013* *1* *; Jejeebhoy et al. 2013* *2* *). Domestic violence is widespread, with one-third of married women reporting an experience; it is, moreover, widely justified, with half (52%) of women and two-fifths (42%) of men justifying violence for at least one of a host of reasons, including going out without permission, neglecting the household and disrespecting in-laws (International Institute for Population Sciences (IIPS) and ICF, 2017* *3* *). In this hierarchical setting, it is likely that men and women will respond differently to the items on the scale developed in this study, for example, embarrassment to declare engagement in violence prevention work, or attitudes about whether protests are effective in stopping violence, and differences would emerge in perceived legitimacy, efficacy and social norms, as well as social desirability responses. Even if the sample size does not permit a sub-group analysis, it seems worthwhile to conduct the analysis separately for men and women to observe any key disparities, with regard to both the scale as well as the correlates.*

We fully agree that it is key to look at gender differences in how people respond to the scale. We have divided this task into three sub-tasks: looking at gender differences in the make-up of factor structure, looking at gender differences in the response to social desirability items, and looking at gender differences in convergent and criterion validity.

Gender differences in factor structure:

We conducted measurement invariance tests across gender, education, caste and marital status and found little evidence for differences in model fit by these groups. The table is displayed in the Results section of the revised manuscript. We added the following text:

We did not find strong evidence against measurement invariance (Table 7). Comparing fit across models accounting for gender, education, caste, and marital status, both WRMR and TLI decreased slightly as constraints on factor loadings were lifted, while RMSEA stayed relatively constant. The decrease in WRMR indicates better fit for models allowing for heterogeneity across groups, but the decrease in TLI indicates a worse fit, possibly due to lower parsimony in the unconstrained models. Given these results, we did not see a need for separate measurement models by gender or demographic group.

(from Section: Results – construct validity)

Gender differences in social desirability:

We have also tested for interactions with these variables in measuring social desirability bias. Here, we find little evidence that female respondents were more strongly affected by social desirability bias than male respondents. The only item for which we found a significant interaction showed men displaying greater social desirability effects than women:

With respect to gender differences in social desirability, we found no evidence for an interaction with gender in all indicators (p>0.05) except for the item “Your family members approve of you joining activities to stop violence against women” (p=0.008), where the self-administered interview had a clear effect on men’s odds of agreeing (OR 0.51, p=0.037, 95% CI 0.27-0.97), but not women’s odds (OR 1.36, p=0.20, 95% 0.85-2.19).

(from Section: Results – item validity)

Gender differences in associations between psychological constructs and behavioural outcomes:

As mentioned above, we wanted the primary focus of the paper to be on measurement and look into substantive issues in forthcoming papers. While it is possible, even likely, that men and women will show different associations between different psychological drivers and participation in collective action, we do not have strong enough evidence to make a priori hypotheses which could inform us if the patterns observed in the data indicate high or low validity for our scale. As such, we think this issue is better explored in a separate paper looking at unpacking these associations in more depth, which we are currently preparing.

*Authors themselves raise questions about their respondents’ understanding of the term ‘violence against women’ and note that they required to explain the term to respondents in some instances. Given that the whole study focuses on violence against women, this possible ambiguity in understanding of the term is cause for concern. Both the World Health Organisation (World Health Organisation, 2005)* *4* *and the Demographic and Health Surveys (see, for example, Kishor and Johnson, 2004)* *5* *have recognised this ambiguity, and have posed, instead of a single question, a battery of questions relating to different acts of physical violence, as well as sexual and emotional violence. Both research programmes recognise the need to specifically define various acts of violence precisely so that the violence measure is not affected by different understandings of what constitutes violence. In India, many associate the term ‘violence’ with extreme practices such as severe beating that results in injury, choking and so on; a slap is an everyday event that few would consider violence, and few would agree that participation in action to counter such an act is warranted. Authors describe steps taken if the respondent asked for clarification, but it is likely that others who did not request clarification may have had a limited definition of violence. An introduction that defines what is implied by the term and applied to all respondents may have reduced these ambiguities in terminology. While this limitation cannot be addressed, it would be helpful to highlight this limitation at greater length, and speculate about possible effects on the scale or other outcomes.*

Thank you for raising this issue. We agree that it is important to reference the WHO and DHS recommendations and have added these points to further emphasise this limitation. Reviewer 2 was also unclear about the main message of this paragraph, so we have extensively rewritten it to clarify:

Our scale also relied on asking people whether they were willing to engage in activism to address ‘violence against women’ ( *mahila ke khilaaf hinsa*) or ‘domestic violence’ ( *gharelu hinsa*). The World Health Organization and the Demographic Health Surveys recommend avoiding the term ‘violence’ wherever possible as there is considerable individual variation in respondent interpretation of the term, including a tendency to consider only extreme practices (e.g. beating or choking) forms of violence, whilst ignoring ‘milder’ practices (e.g. slapping) [INSERT REFERENCES 65 & 66]. However, in a survey setting, it would be unreasonably unwieldy to ask separate questions for attitudes to action against slapping, attitudes to action against kicking, attitudes to action against forced sex, etc. Global health researchers thus universally invoke the generic term ‘violence’ in questions on participation in action to address VAW ^39^^, ^^55^ , as do researchers on bystander intervention ^56^ . However, this practice does cause confusion: respondents sometimes asked during interviews what the word ‘violence’ meant, which required interviewers to clarify. We piloted alternatives for the word ‘violence’, but these created even more confusion – ‘forcing/coercing’ someone ( *zabardasti karna)* has the unfortunate alternate sense of ‘insisting on something’, while the word ‘force’/ *bal *does not have an intrinsic negative valence like the word ‘violence’ and may even have positive connotations of ‘strength’ and ‘power’. In future research, there may be merit in exploring more blunt phrases such as ‘action to stop husbands beating their wives’ as proxies for ‘action to address violence’. During piloting, respondents said it was hard to distinguish collective action against different forms of VAW, as physical, sexual, and emotional violence tended to occur together for survivors of violence.

(from Section: Discussion)

*Given the limited voice that women and girls exercise in families in India, many personal questions can raise discomfort levels among them. Hence, typically, sections of surveys dealing with these issues are conducted in private, without mothers and mothers-in-law listening in. Without an assurance of privacy, it is very possible that women’s responses would be more likely to adhere to traditional norms and expectations than if privacy is ensured. In this case, while it is true that personal experiences are not elicited, attitudes about the acceptability of intervention and collective action or perceptions about how families would react to their participation in collective action to stop a behaviour that is perceived as so acceptable, may also cause discomfort to young women who are audible to their mothers-in-law, and result in responses that are acceptable to those listening in. While here too, nothing can be done now, it would be helpful to highlight this limitation and offer some ideas about how it might have affected findings.*

Thank you for this point. We have discussed this in connection with social desirability, as our findings did indicate that respondents showed the greatest difference in answers on the question concerning family attitudes and norms. We felt we had already addressed this point in the existing text, so we did not want to add more text.

*Small points. Social capital is discussed, and adjustments made for it, but there is no indication in the paper of the level of social capital in the study sample, it would be good to highlight this or explain why it has been omitted.*

We have added a brief descriptive paragraph about our social capital data to supply this information. We added the precise numbers into a Supplementary Table to preserve readability.

We found high levels of social cohesion in our communities, as 81-93% of respondents reported feeling at home in their neighbourhood or getting along with neighbours (see *Extended data*, Supplementary Table 3 ^20^). 71-91% agreed that people came together to keep the neighbourhood free from crime or solve issues such as disruptions to the water supply. However, levels of trust were lower, as 58-61% stated that their neighbours could not be trusted or only looked out for themselves. 56% of women and 46% of men stated they did not recognise most people in their neighbourhood (p<0.001 for a gender difference).

(from Section: Results – Descriptive statistics)

*Also, authors may want to think of a friendlier way of displaying the information provided in Figure 3.*

Thank you for this point. We experimented with displaying Figure 3 as a table previously, but felt ultimately the Figure was easier to read than a table.

**References**

1.           Gram, L., N. Daruwalla, and D. Osrin, *Understanding participation dilemmas in community mobilisation: can collective action theory help?* J Epidemiol Community Health, 2019. **73**(1): p. 90-96.

2.           Gram, L., et al., *Promoting women and children's health through community-based groups in low- and middle-income countries: a mixed methods systematic review of mechanisms, enablers and barriers.* BMJ Global Health, 2019. **4**: p. e001972.

3.           Van Zomeren, M., T. Postmes, and R. Spears, *Toward an integrative social identity model of collective action: a quantitative research synthesis of three socio-psychological perspectives.* Psychological Bulletin, 2008. **134**(4): p. 504.

4.           Ostrom, E., *A behavioral approach to the rational choice theory of collective action: Presidential address, American Political Science Association, 1997.* American Political Science Review, 1998. **92**(1): p. 1-22.

5.           Fokkema, M. and S. Greiff, *How performing PCA and CFA on the same data equals trouble*. 2017, Hogrefe Publishing.

6.           Clark, C.J., et al., *Social norms and women's risk of intimate partner violence in Nepal.* Social Science & Medicine, 2018. **202**: p. 162-169.

7.           Ostrom, E., *Collective action and the evolution of social norms.* Journal of Natural Resources Policy Research, 2014. **6**(4): p. 235-252.

8.           Kahneman, D., *Maps of bounded rationality: Psychology for behavioral economics.* The American Economic Review, 2003. **93**(5): p. 1449-1475.

9.           Henrich, J. and R. McElreath, *Dual inheritance theory: the evolution of human cultural capacities and cultural evolution.* Oxford handbook of evolutionary psychology, 2007: p. 555-570.

10.        Andreoni, J. and J.H. Miller, *Analyzing choice with revealed preference: is altruism rational?* Handbook of experimental economics results, 2008. **1**: p. 481-487.

11.        Heise, L.L., *Violence against women an integrated, ecological framework.* Violence against women, 1998. **4**(3): p. 262-290.

12.        Lippman, S.A., et al., *Conceptualizing community mobilization for HIV prevention: implications for HIV prevention programming in the African context.* PloS one, 2013. **8**(10): p. e78208.

13.        Gram, L., J. Morrison, and J. Skordis-Worrall, *Organising concepts of ‘women’s empowerment’ for measurement: a typology.* Social Indicators Research, 2018: p. 1-28.

14.        King, G., et al., *Enhancing the validity and cross-cultural comparability of measurement in survey research.* American Political Science Review, 2004. **98**(1): p. 191-207.

15.        Gram, L., et al., *Validating an Agency-Based Tool for Measuring Women's Empowerment in a Complex Public Health Trial in Rural Nepal.* Journal of Human Development and Capabilities, 2016: p. 1-29.

16.        Abbasi, M., et al., *A cross-sectional study examining convergent validity of a frailty index based on electronic medical records in a Canadian primary care program.* BMC geriatrics, 2019. **19**(1): p. 109.

17.        Ljótsson, B., et al., *Discriminant and convergent validity of the GSRS-IBS symptom severity measure for irritable bowel syndrome: A population study.* United European Gastroenterology Journal, 2020. **8**(3): p. 284-292.

18.        Daruwalla, N., et al., *Community interventions to prevent violence against women and girls in informal settlements in Mumbai: the SNEHA-TARA pragmatic cluster randomised controlled trial.* Trials, 2019. **In Press**.

# Peer review: Reviewer response for version 1

**DOI:** 10.21956/wellcomeopenres.17216.r38811

**Published:** 2020-05-29

**Authors:** Andrew Gibbs

**Affiliations:** 1 Gender and Health Research Unit, South African Medical Research Council, Pretoria, South Africa

Overall, this is an interesting, and novel approach to understanding collective action around violence against women and girls, given that much has been written about it, but little has been assessed quantitatively. I generally have only a few concerns.

1. I wonder if a note around limitations is needed about the fact 30% could not answer one item (or more), and the implications of this for the scale. Also, did this vary by administration technique?
2. On page 9, the first mention of "clinically significant" appears, without really clarifying what this means (in terms of scales). Could this be clarified.
3. In the discussion, it is stated that an important thing for community activists to do would be to shift from persuading residents of the wrongness of VAW towards engaging them around their efficacy and normative beliefs. I think this is a key point that gets lost a little, as it's an important thing to consider in terms of intervention development, and it somewhat gets lost.
4. Closely linked, is potentially the need to think a little more of the implications of the analysis for social norms theory. Social norms are briefly mentioned at the start of the paper, and it would be worth returning to this concept again in the discussion. In some ways, based on the point above, the authors may be suggesting social norms are not the main issue, but rather the need to support people to recognise they can make a difference. A reflection on social norms theory, would therefore be useful. 
5. I did not fully get, on page 14, the sentence: "In a survey setting, it is generally not possible to ask detailed questions about actions taken to address individual acts of violence (slapping, kicking..." the list read like the questions about IPV you would ask. I think a few additional words to explain what is meant would be helpful.

Generally, I found the paper well written and a useful contribution.

Is the work clearly and accurately presented and does it cite the current literature?

Yes

If applicable, is the statistical analysis and its interpretation appropriate?

Yes

Are all the source data underlying the results available to ensure full reproducibility?

Yes

Is the study design appropriate and is the work technically sound?

Yes

Are the conclusions drawn adequately supported by the results?

Yes

Are sufficient details of methods and analysis provided to allow replication by others?

Yes

Reviewer Expertise:

Community mobilisation, violence against women.

I confirm that I have read this submission and believe that I have an appropriate level of expertise to confirm that it is of an acceptable scientific standard.

# Response

**Published:** 2020-06-23

**Authors:** Lu Gram

**Affiliations:** University College London, UK

Thank you so much for your constructive feedback. We have replied point-by-point below:

*I wonder if a note around limitations is needed about the fact 30% could not answer one item (or more), and the implications of this for the scale.*

This is a good suggestion. We have added a note in the limitations section.

Finally, our study was limited by missing data, as 30% of respondents lacked data for at least one indicator. However, at most 8% of respondents did not know the answer to any single indicator. As stated earlier, we used complete case analysis for item validity on an item-by-item basis, while we used imputation to create an index of items to assess criterion validity. As respondents with missing data were more likely to be poorly educated, this may have upwardly biased assessments of scale validity.

(from Section: Discussion)

*Also, did this vary by administration technique?*

You are right that it would be useful to check for this. There were indications that the self-administered technique produced slightly more “don’t know” answers. This might either be due to respondents feeling more comfortable admitting to not knowing the answer in the self-administered condition, or because the technique was more difficult for them to use. We have added detail on this to the Results and discussed in the Discussion:

The proportion of respondents providing “don’t know” answers generally increased by 1-4 percentage points in the self-administered survey condition (p<0.05). However, for the item “People in your neighbourhood approve of you joining activities to stop violence against women”, the proportion of “don’t know” answers increased by fully 6 pp (p=0.005).

(from Section: Results – Item validity)

Some respondents may have had difficulty navigating the mobile phone technology on their own, as the proportion of “don’t know” answers increased in the self-administered condition. However, this may also reflect respondents being more comfortable voicing genuine ignorance in self-administered than in to face-to-face interviews.

(from Section: Discussion)

*On page 9, the first mention of "clinically significant" appears, without really clarifying what this means (in terms of scales). Could this be clarified.*

Thank you for this comment. We agree the term is misleading and it would be simpler to just state the relevant magnitudes. We have changed the phrase “[these differences] were not clinically significant” to “[these differences] seem small”, followed by just stating the magnitudes.

*In the discussion, it is stated that an important thing for community activists to do would be to shift from persuading residents of the wrongness of VAW towards engaging them around their efficacy and normative beliefs. I think this is a key point that gets lost a little, as it's an important thing to consider in terms of intervention development, and it somewhat gets lost.*

We agree that this is an important point to make. We are a little hesitant to further draw out the point: as pointed out by Reviewer 3, there is a distinction between a paper seeking to validate a new scale and a paper seeking to draw new substantive conclusions about the world. In this paper, we only looked briefly at associations with behavioural measures to establish convergent validity and strengthen the plausibility of our scale. To truly unpack the complex relationships between psychological drivers of collective action, participation in such action, and domestic violence, we would need to take into account a host of issues (confounding, reverse causality and effect modification) that fit better into a separate paper. We are currently in the process of writing this paper and will definitely take your advice into account in framing its conclusions. We have now added a caveat about this:

These findings show the utility of our scale, although further research is required to fully disentangle these complex relationships.

(from Section: Discussion)

*Closely linked, is potentially the need to think a little more of the implications of the analysis for social norms theory. Social norms are briefly mentioned at the start of the paper, and it would be worth returning to this concept again in the discussion. In some ways, based on the point above, the authors may be suggesting social norms are not the main issue, but rather the need to support people to recognise they can make a difference. A reflection on social norms theory, would therefore be useful.*

This is an interesting suggestion. In line with our point above, we are wary about adding too much reflection on substantive implications of this research. However, we will certainty include consideration of social norms theory in our follow-up paper on substantive issues.

*I did not fully get, on page 14, the sentence: "In a survey setting, it is generally not possible to ask detailed questions about actions taken to address individual acts of violence (slapping, kicking..." the list read like the questions about IPV you would ask. I think a few additional words to explain what is meant would be helpful.*

Thank you for asking this question; it made us realise that the paragraph was unclearly rewritten. We have extensively rewritten the paragraph, so that the message is clearer.

Our scale also relied on asking people whether they were willing to engage in activism to address ‘violence against women’ ( *mahila ke khilaaf hinsa*) or ‘domestic violence’ ( *gharelu hinsa*). The World Health Organization and the Demographic Health Surveys recommend avoiding the term ‘violence’ wherever possible as there is considerable individual variation in respondent interpretation of the term, including a tendency to consider only extreme practices (e.g. beating or choking) forms of violence, whilst ignoring ‘milder’ practices (e.g. slapping) [INSERT REFERENCES 65 & 66]. However, in a survey setting, it would be unreasonably unwieldy to ask separate questions for attitudes to action against slapping, attitudes to action against kicking, attitudes to action against forced sex, etc. Global health researchers thus universally invoke the generic term ‘violence’ in questions on participation in action to address VAW ^39^^, ^^55^ , as do researchers on bystander intervention ^56^ . However, this practice does cause confusion: respondents sometimes asked during interviews what the word ‘violence’ meant, which required interviewers to clarify. We piloted alternatives for the word ‘violence’, but these created even more confusion – ‘forcing/coercing’ someone ( *zabardasti karna)* has the unfortunate alternate sense of ‘insisting on something’, while the word ‘force’/ *bal *does not have an intrinsic negative valence like the word ‘violence’ and may even have positive connotations of ‘strength’ and ‘power’. In future research, there may be merit in exploring more blunt phrases such as ‘action to stop husbands beating their wives’ as proxies for ‘action to address violence’. During piloting, respondents said it was hard to distinguish collective action against different forms of VAW, as physical, sexual, and emotional violence tended to occur together for survivors of violence.

(from Section: Discussion)

**References**

1.           Gram, L., N. Daruwalla, and D. Osrin, *Understanding participation dilemmas in community mobilisation: can collective action theory help?* J Epidemiol Community Health, 2019. **73**(1): p. 90-96.

2.           Gram, L., et al., *Promoting women and children's health through community-based groups in low- and middle-income countries: a mixed methods systematic review of mechanisms, enablers and barriers.* BMJ Global Health, 2019. **4**: p. e001972.

3.           Van Zomeren, M., T. Postmes, and R. Spears, *Toward an integrative social identity model of collective action: a quantitative research synthesis of three socio-psychological perspectives.* Psychological Bulletin, 2008. **134**(4): p. 504.

4.           Ostrom, E., *A behavioral approach to the rational choice theory of collective action: Presidential address, American Political Science Association, 1997.* American Political Science Review, 1998. **92**(1): p. 1-22.

5.           Fokkema, M. and S. Greiff, *How performing PCA and CFA on the same data equals trouble*. 2017, Hogrefe Publishing.

6.           Clark, C.J., et al., *Social norms and women's risk of intimate partner violence in Nepal.* Social Science & Medicine, 2018. **202**: p. 162-169.

7.           Ostrom, E., *Collective action and the evolution of social norms.* Journal of Natural Resources Policy Research, 2014. **6**(4): p. 235-252.

8.           Kahneman, D., *Maps of bounded rationality: Psychology for behavioral economics.* The American Economic Review, 2003. **93**(5): p. 1449-1475.

9.           Henrich, J. and R. McElreath, *Dual inheritance theory: the evolution of human cultural capacities and cultural evolution.* Oxford handbook of evolutionary psychology, 2007: p. 555-570.

10.        Andreoni, J. and J.H. Miller, *Analyzing choice with revealed preference: is altruism rational?* Handbook of experimental economics results, 2008. **1**: p. 481-487.

11.        Heise, L.L., *Violence against women an integrated, ecological framework.* Violence against women, 1998. **4**(3): p. 262-290.

12.        Lippman, S.A., et al., *Conceptualizing community mobilization for HIV prevention: implications for HIV prevention programming in the African context.* PloS one, 2013. **8**(10): p. e78208.

13.        Gram, L., J. Morrison, and J. Skordis-Worrall, *Organising concepts of ‘women’s empowerment’ for measurement: a typology.* Social Indicators Research, 2018: p. 1-28.

14.        King, G., et al., *Enhancing the validity and cross-cultural comparability of measurement in survey research.* American Political Science Review, 2004. **98**(1): p. 191-207.

15.        Gram, L., et al., *Validating an Agency-Based Tool for Measuring Women's Empowerment in a Complex Public Health Trial in Rural Nepal.* Journal of Human Development and Capabilities, 2016: p. 1-29.

16.        Abbasi, M., et al., *A cross-sectional study examining convergent validity of a frailty index based on electronic medical records in a Canadian primary care program.* BMC geriatrics, 2019. **19**(1): p. 109.

17.        Ljótsson, B., et al., *Discriminant and convergent validity of the GSRS-IBS symptom severity measure for irritable bowel syndrome: A population study.* United European Gastroenterology Journal, 2020. **8**(3): p. 284-292.

18.        Daruwalla, N., et al., *Community interventions to prevent violence against women and girls in informal settlements in Mumbai: the SNEHA-TARA pragmatic cluster randomised controlled trial.* Trials, 2019. **In Press**.

# Peer review: Reviewer response for version 1

**DOI:** 10.21956/wellcomeopenres.17216.r37882

**Published:** 2020-05-27

**Authors:** Kathryn M. Yount

**Affiliations:** 1 Hubert Department of Global Health ad Department of Sociology, Emory University, Atlanta, GA, USA

This manuscript is an important contribution to the literature on understanding the conditions that drive participation in collective action to address violence against women in a setting where such violence is prevalent. Below are questions/suggestions that may improve the contributions of this manuscript.

1. Did the authors conduct an expert review of question items to operationalize the psychological drivers of collective action.
2. In the authors' pilot of the questionnaire, did they conduct formal cognitive interviews to assess the potential sources of response bias?
3. In the analysis, why did the authors choose not to conduct an exploratory factor analysis of the items before conducting a confirmative factor analysis? An EFA normally is recommended when new constructs are being measured with little empirical evidence of their validity. I encourage the authors to consider EFA first to refine the measurement model, and then CFA.
4. Did the authors assess the measurement invariance of the constructs of interest across theoretically relevant subgroups in the study site. Some demonstration of within-sample measurement invariance would strengthen the claim that the measures developed are useful across diverse and theoretically relevant populations of interest.
5. I am concerned about the absence of a relation between social capital (a resource for collective action) and participation in collective action. I encourage the authors to discuss in greater detail their measure of social capital.
6. Some reference to the theoretical literature on women's empowerment is warranted. how to resources for women's agency, including collective agency, factor into the conceptualization of this analysis?

Thank you for the opportunity to review this important manuscript. I wish the authors all the best in this important work.

Is the work clearly and accurately presented and does it cite the current literature?

Yes

If applicable, is the statistical analysis and its interpretation appropriate?

Partly

Are all the source data underlying the results available to ensure full reproducibility?

Yes

Is the study design appropriate and is the work technically sound?

Partly

Are the conclusions drawn adequately supported by the results?

Partly

Are sufficient details of methods and analysis provided to allow replication by others?

Partly

Reviewer Expertise:

Measurement; violence against women prevention.

I confirm that I have read this submission and believe that I have an appropriate level of expertise to confirm that it is of an acceptable scientific standard, however I have significant reservations, as outlined above.

# Response

**Published:** 2020-06-23

**Authors:** Lu Gram

**Affiliations:** University College London, UK

Thank you so much for your constructive suggestions. We have implemented the suggestions as far as possible and feel that this has improved the manuscript.

*Did the authors conduct an expert review of question items to operationalize the psychological drivers of collective action.*

We solicited feedback on our theoretical framework from experts on collective action, but we did not conduct a formal expert review due to time constraints. The lead author LG was involved in the overall randomized controlled trial just as baseline data collection was starting (hence the missing data for first two trial clusters), so there was not time for formal review. However, we reviewed literature on social movements, collective action, and community mobilisation prior to designing survey items [1, 2]. We have clarified this now:

We conducted a literature review of social movements, collective action and community mobilisation to inform choice of indicators [INSERT REFERENCES 4 & 9]. We did not conduct a formal expert review.

(from Section: Indicator Selection) 

*In the authors' pilot of the questionnaire, did they conduct formal cognitive interviews to assess the potential sources of response bias?*

We did not conduct formal cognitive interviews, as the above time constraints prevented us from recording, translating and coding transcripts. However, the nature of the questions in our survey were such that respondents usually provided whole sentences or short anecdotes in response to them, which we used as a starting point for further discussion of their understanding of our questions. In the few cases in which respondents only answered “yes” or “no” to our questions, we probed by asking them to explain the reason behind their answer and how it might relate to answers they had provided to other questions during the interview. We have now clarified this:

These were not formal cognitive interviews [INSERT REFERENCE 58], but organic interactions emerging from respondents providing short anecdotes or observations in response to survey items. If respondents only answered “yes” or “no” to our questions, LG probed for the reason behind their answer to stimulate discussion.

(from Section: Piloting)

*In the analysis, why did the authors choose not to conduct an exploratory factor analysis of the items before conducting a confirmative factor analysis? An EFA normally is recommended when new constructs are being measured with little empirical evidence of their validity. I encourage the authors to consider EFA first to refine the measurement model, and then CFA.*

Thank you for this suggestion. We saw the situation slightly differently, as the overall constructs have been explored extensively outside the context of preventing violence against women in low- and middle-income contexts [3, 4]. Some social psychologists and anthropologists whom we consulted even thought it redundant to test these constructs in the context of VAW. We do believe evidence is needed, as past studies have primarily taken place in high-income contexts on issues unrelated to violence against women or health more broadly, but we used CFA rather than EFA because:

- We were testing constructs that had already been proposed elsewhere.
- We were unsure how to compare the results of EFA and CFA given the fact that hierarchical CFA imposes a number of identifiability restrictions that do not have equivalents in EFA regarding the relation between lower- and higher-order factors.
- Given that EFA and CFA on the same dataset is generally not recommended to avoid overfitting [5], we were also unsure about how to use the results of EFA post hoc now that we have conducted our CFA.

*Did the authors assess the measurement invariance of the constructs of interest across theoretically relevant subgroups in the study site. Some demonstration of within-sample measurement invariance would strengthen the claim that the measures developed are useful across diverse and theoretically relevant populations of interest.*

This is a good point. We have added measurement invariance tests across gender, education, caste  and marital status now. Please see the new table in the results section of the updated manuscript.

While RMSEA stays nearly constant (up to rounding errors), WRMR reduces with fewer parameter restrictions, indicating a better model fit. However, TLI also reduces with fewer parameter restrictions, indicating a worse fit – possibly due to TLI penalizing lack of parsimony – as we go from scalar invariant to configural invariant models. All in all, the changes in TLI, RMSEA and WRMR seem small and all models have good fit statistics on the TLI and RMSEA by conventional criteria. Given these results, we did not feel justified in using separate models by gender, education, caste or marital status in estimating our latent constructs. We have now added this information in the text itself:

Finally, we assessed measurement invariance across gender, education, caste, and marital status by comparing fit statistics (TLI, RMSEA, and WRMR) for models of configural invariance, invariance of first-order factor loadings, invariance of second-order factor loadings, and invariance of both [INSERT REFERENCE 59]. For the purpose of this comparison, we used binary codes for education (any versus no education), caste (Open/General versus other caste groups), and marital status (currently married versus not currently married). We did not conduct χ2 tests as these are known to be overly dependent on sample size, which creates oversensitivity to small deviations from H _0_ in large samples and spurious sensitivity to differences in sample size between comparison groups in measurement invariance tests [INSERT REFERENCE 60].

(from Section: Data analysis – construct validity)

We did not find strong evidence against measurement invariance (Table 7). Comparing fit across models accounting for gender, education, caste, and marital status, both WRMR and TLI decreased slightly as constraints on factor loadings were lifted, while RMSEA stayed relatively constant. The decrease in WRMR indicates better fit for models allowing for heterogeneity across groups, but the decrease in TLI indicates a worse fit, possibly due to lower parsimony in the unconstrained models. Given these results, we did not see a need for separate measurement models by gender or demographic group.

(from Section: Results – construct validity)

*I am concerned about the absence of a relation between social capital (a resource for collective action) and participation in collective action. I encourage the authors to discuss in greater detail their measure of social capital.*

Thank you for highlighting this. We realise our wording in the Results section has been misleading: while we did not find a direct association between social capital and participation in collective action after adjusting for potential mediators, we did find an indirect association: social capital increased mediator variables, which in turn increased participation. We have added a table with indirect effects of social capital to clarify this (see main document) and also changed the text:

Social capital was itself positively associated with perceived legitimacy, perceived efficacy, and collective action norms (p<0.001). There was insufficient evidence that social capital was directly associated with participation in groups to address VAW and intent to intervene on behalf of a family member (p>0.05) after adjusting for mediators. Social capital itself was directly negatively associated with intent to intervene on behalf of a stranger, showing a 27-30% reduction in odds of intervening with one standard deviation increase in social capital. However, the indirect effect of social capital on outcomes through perceived legitimacy, perceived efficacy, and collective action norms was positive in all cases (Table 8). Overall, these results suggest that our three constructs did not simply predict outcomes due to their association with social capital.

(from Section: Results – criterion validity)

The relationship between social capital and intervention in domestic violence is likely complex. Greater social cohesion can act as a resource for collective action to prevent VAW, e.g. fostering collective action norms rewarding participation in such action, and it may increase perceived efficacy as community members feel their close links with neighbours empower them to overcome common threats. However, cohesive communities led by male community leaders may also exert stronger patriarchal control over women. As a validation paper, we did a first check to see if the scale produces plausible evidence for convergent and discriminant validity. We plan to unpack these complex relationships in greater depth in future papers on substantive theory. We have now clarified this:

We found no evidence for social capital being positively associated with participation in collective action after adjusting for psychological drivers. Past evidence paints a mixed picture of the role of general social capital in preventing domestic violence, as studies have found it variously beneficial [INSERT REFERENCE 61], harmful [INSERT REFERENCE 63], or neutral [INSERT REFERENCE 62]. Although community-level social capital may increase access to support networks for women, it may also empower male community members to police women’s use of such networks [INSERT REFERENCE 64]. Social norms disapproving of violence may also be required to translate social capital into action: a trial of a violence prevention programme in Uganda found that social capital was only associated with bystander intervention in intervention areas, not control areas ^39^ . We even found that social capital was negatively associated with intent to intervene in case of VAW against a stranger. This echoes literature on the ‘dark side of social capital’ ^45^ , which suggests that tightly connected social networks can be detrimental to the health of perceived outsiders by excluding them from the support of network insiders. However, further research is required to unpack the relationship between social capital and VAW.

(from Section: Discussion)

We were a little unsure as to what additional detail is needed for our measure of social capital. In case it has to do with choice of indicators, we have clarified that these are now all spelled out in full in the extended data.

( *Extended data*, Supplementary Table 1 ^20^ lists survey items for main and complementary measures in full).

(from Section: Indicator Selection)

*Some reference to the theoretical literature on women's empowerment is warranted. how to resources for women's agency, including collective agency, factor into the conceptualization of this analysis?*

We fully agree that reflection on the relationship between our work and women’s empowerment is warranted. We have now discussed this:

Promoting women’s capacity for collective action to address VAW also contributes to global policy commitments to advance gender equality and women’s empowerment [INSERT REFERENCE 67]. Researchers have extensively studied individualistic conceptions of agency and empowerment using measures of household decision-making power, spousal bargaining power, and access to material resources [INSERT REFERENCES 68-71]. However, women’s collective agency and empowerment remain poorly understood, and few quantitative measures are available [INSERT REFERENCE 72]. Group participation [INSERT REFERENCE 73] and social network structure [INSERT REFERENCE 74] have been used in the past, but such proxies preclude researchers from separating out capacity for collective action from participation in such action. Groups and associations are often dynamic social structures, which form, disband or evolve based on perceived need [INSERT REFERENCE 75], thus capacity for collective action may be just as important to track as actual participation.

(from Section: Introduction)

**References**

1.           Gram, L., N. Daruwalla, and D. Osrin, *Understanding participation dilemmas in community mobilisation: can collective action theory help?* J Epidemiol Community Health, 2019. **73**(1): p. 90-96.

2.           Gram, L., et al., *Promoting women and children's health through community-based groups in low- and middle-income countries: a mixed methods systematic review of mechanisms, enablers and barriers.* BMJ Global Health, 2019. **4**: p. e001972.

3.           Van Zomeren, M., T. Postmes, and R. Spears, *Toward an integrative social identity model of collective action: a quantitative research synthesis of three socio-psychological perspectives.* Psychological Bulletin, 2008. **134**(4): p. 504.

4.           Ostrom, E., *A behavioral approach to the rational choice theory of collective action: Presidential address, American Political Science Association, 1997.* American Political Science Review, 1998. **92**(1): p. 1-22.

5.           Fokkema, M. and S. Greiff, *How performing PCA and CFA on the same data equals trouble*. 2017, Hogrefe Publishing.

6.           Clark, C.J., et al., *Social norms and women's risk of intimate partner violence in Nepal.* Social Science & Medicine, 2018. **202**: p. 162-169.

7.           Ostrom, E., *Collective action and the evolution of social norms.* Journal of Natural Resources Policy Research, 2014. **6**(4): p. 235-252.

8.           Kahneman, D., *Maps of bounded rationality: Psychology for behavioral economics.* The American Economic Review, 2003. **93**(5): p. 1449-1475.

9.           Henrich, J. and R. McElreath, *Dual inheritance theory: the evolution of human cultural capacities and cultural evolution.* Oxford handbook of evolutionary psychology, 2007: p. 555-570.

10.        Andreoni, J. and J.H. Miller, *Analyzing choice with revealed preference: is altruism rational?* Handbook of experimental economics results, 2008. **1**: p. 481-487.

11.        Heise, L.L., *Violence against women an integrated, ecological framework.* Violence against women, 1998. **4**(3): p. 262-290.

12.        Lippman, S.A., et al., *Conceptualizing community mobilization for HIV prevention: implications for HIV prevention programming in the African context.* PloS one, 2013. **8**(10): p. e78208.

13.        Gram, L., J. Morrison, and J. Skordis-Worrall, *Organising concepts of ‘women’s empowerment’ for measurement: a typology.* Social Indicators Research, 2018: p. 1-28.

14.        King, G., et al., *Enhancing the validity and cross-cultural comparability of measurement in survey research.* American Political Science Review, 2004. **98**(1): p. 191-207.

15.        Gram, L., et al., *Validating an Agency-Based Tool for Measuring Women's Empowerment in a Complex Public Health Trial in Rural Nepal.* Journal of Human Development and Capabilities, 2016: p. 1-29.

16.        Abbasi, M., et al., *A cross-sectional study examining convergent validity of a frailty index based on electronic medical records in a Canadian primary care program.* BMC geriatrics, 2019. **19**(1): p. 109.

17.        Ljótsson, B., et al., *Discriminant and convergent validity of the GSRS-IBS symptom severity measure for irritable bowel syndrome: A population study.* United European Gastroenterology Journal, 2020. **8**(3): p. 284-292.

18.        Daruwalla, N., et al., *Community interventions to prevent violence against women and girls in informal settlements in Mumbai: the SNEHA-TARA pragmatic cluster randomised controlled trial.* Trials, 2019. **In Press**.
